# Social, emotional, and personality factors shape four psychological well‐being profiles: A clustering approach in young adults with affinity propagation algorithm

**DOI:** 10.1111/aphw.70072

**Published:** 2025-08-29

**Authors:** Assunta Pelagi, Chiara Camastra, Alessia Sarica

**Affiliations:** ^1^ Neuroscience Research Center, Department of Medical and Surgical Sciences Magna Graecia University Catanzaro Italy

**Keywords:** affinity propagation, clustering, cognitive functioning, personality traits, psychological well‐being

## Abstract

Psychological well‐being (PWB) is a multidimensional construct encompassing emotional, cognitive, personality, and social factors, playing a crucial role in mental health and quality of life. While previous research has examined the relationships between PWB and psychological traits, the natural clustering of well‐being profiles remains underexplored.

This study applied Affinity Propagation (AP) clustering, an unsupervised machine learning (ML) technique, to identify distinct well‐being profiles in 685 young adults from the Human Connectome Project (HCP). A composite PWB score from the NIH Toolbox Emotion Battery was used to assess its associations with cognitive functions, personality traits, emotional health, and psychiatric and behavioral factors.

Four PWB clusters emerged: Low, Medium‐low, Medium‐high, and High. Lower PWB was linked to higher negative affect (anger, sadness) and greater neuroticism, while higher social support, extraversion, agreeableness, and conscientiousness characterized greater well‐being. Cognitive abilities did not significantly differentiate clusters, suggesting well‐being is primarily influenced by emotional, social, and personality factors.

By integrating ML with statistical analyses, this study provides a data‐driven understanding of well‐being, emphasizing the need for targeted interventions to enhance emotional resilience, social connections, and mental health support.

## INTRODUCTION

Psychological well‐being (PWB) is a multifaceted construct encompassing emotional, cognitive, and social dimensions, making it a cornerstone of mental health and public health initiatives (Organization, 2003). As a key determinant of resilience and quality of life, PWB plays a pivotal role in shaping mental health outcomes and informing interventions designed to enhance well‐being (Diener et al., 1999). Theoretical frameworks distinguish between two primary perspectives on well‐being: the hedonic approach, which emphasizes pleasure, happiness, and life satisfaction (Ryan & Deci, 2003), and the eudaimonic approach, which focuses on purpose, personal growth, and self‐actualization (Ryff, 1989). While hedonia is closely linked to subjective well‐being, eudaimonia aligns with the broader concept of psychological well‐being (Santisi et al., 2020). These perspectives have significantly shaped research, demonstrating that well‐being fosters adaptive behaviors, including effective stress management and strong social engagement (Waterman, 2008).

Higher levels of PWB have been associated with better physical health, lower risks of chronic illness, and increased longevity (Huppert & Whittington, 2003). Conversely, reduced well‐being has been linked to psychological distress, including heightened anxiety and depressive symptoms (Kleftaras & Psarra, 2012). Given these far‐reaching implications, understanding the factors that contribute to different levels of well‐being is critical for both theoretical exploration and the development of effective psychological interventions. However, assessing PWB presents methodological challenges due to its dynamic and complex nature, which is influenced by environmental, social, and individual characteristics (Huppert, 2009). This complexity necessitates the use of innovative analytical approaches that can capture the multidimensionality of well‐being beyond traditional linear associations (Huppert, 2017).

Existing research has examined how personality traits, emotional functioning, and cognitive abilities relate to PWB (Jokela, 2022; Joshanloo, 2023; Kokko et al., 2013; Yu et al., 2024), but most studies have relied on predefined categories or linear relationships, potentially overlooking hidden patterns that naturally differentiate individuals based on their well‐being experiences. The natural clustering of these psychological dimensions could be explored through unsupervised learning, enhancing our ability to understand psychological well‐being heterogeneity fully (Mahon et al., 2022; Orchard et al., 2024).

In a previous study, predictive machine learning models, such as Random Forest, were used to examine the most relevant emotions variables in predicting psychological well‐being, focusing on NIHTB‐EB scores (Pelagi et al., 2024). While this approach provided valuable insights into key determinants of well‐being, it was limited by its reliance on predefined relationships, which may not fully capture the complexity and natural variability of psychological well‐being. To build upon these findings, the present study employs an unsupervised clustering approach to investigate how individuals naturally group according to their well‐being profiles.

Several clustering approaches exist, including hierarchical methods, partition‐based methods, and exemplar‐based methods. Hierarchical clustering builds nested groupings by successively merging or splitting data points, offering a visual representation through dendrograms. Partition‐based methods, such as k‐means, divide data into fixed clusters based on distance metrics. For example, clustering techniques have been widely applied in psychological well‐being research. Nishi et al. (2025) used k‐means clustering analysis to examine the relationship between smartphone screen time, psychological well‐being, gender, and age among Japanese adults, identifying distinct user profiles based on device usage patterns. Similarly, Pancheva et al. (2021) employed a clustering‐and‐projection technique known as Self‐Organizing Maps (SOM), an unsupervised neural network approach, to explore the structure of hedonic and eudaimonic well‐being, uncovering meaningful psychological profiles beyond traditional predefined categories. Moreover, Lingelbach et al. (2022) applied an unsupervised clustering method combining Gower's distance with the Partitioning Around Medoids (PAM) algorithm (k‐Medoids) to analyze how psychological well‐being was affected by the COVID‐19 pandemic, revealing subgroups with distinct emotional responses.

However, conventional clustering methods, such as k‐means or hierarchical clustering, require predefined assumptions about the number of clusters, introducing human bias. For this reason, the present study applies Affinity Propagation (AP) clustering, a data‐driven unsupervised machine learning technique (Frey & Dueck, 2007), to identify distinct psychological well‐being profiles among a large cohort from the Human Connectome Project (HCP) Young Adult (Van Essen et al., 2013). AP dynamically determines the optimal number of clusters based on exemplar‐based learning (Sarica et al., 2017; Madhulatha, 2012), and this makes it particularly suitable for psychological research, where the structure of well‐being variations may not be well‐defined (Brusco et al., 2019). Nonetheless, it is essential to recognize that AP also entails a model selection process, similar to the requirement to prespecify the number of clusters in methods such as k‐means. In AP, this decision is reflected in the setting of the preference parameter, which influences the number of clusters derived. Following the recommendation by Frey and Dueck (2007), we set the preference to the minimum similarity value, resulting in a relatively small number of clusters.

AP has been successfully applied across various fields, demonstrating its flexibility in clustering complex data structures where the optimal number of clusters is unknown. For instance, Nagayi and Nyirenda (2024) enhanced AP for public sentiment analysis, integrating it with Agglomerative Hierarchical Clustering to improve text‐based sentiment classification. Likewise, Qian et al. (2024) utilized AP to extract key features in COVID‐19 mortality analysis, identifying clusters of risk factors contributing to increased mortality rates. In the field of neurology, Sarica et al. (2021) applied AP for cognitive clustering in Parkinsonisms, uncovering heterogeneous cognitive profiles among individuals with Parkinson's disease and Progressive Supranuclear Palsy, demonstrating its applicability in understanding complex neuropsychological patterns.

In the present study, we applied the AP algorithm on a derived composite measure of PWB from the National Institute of Health Toolbox Emotion Battery (NIHTB‐EB) (Salsman et al., 2013). This derived score integrates life satisfaction, positive affect, and meaning and purpose into a standardized assessment of well‐being, following the methodology proposed by Babakhanyan et al. (Babakhanyan et al., 2018).

The NIHTB‐EB is a comprehensive, standardized tool developed to assess various dimensions of emotional health, including Psychological Well‐being, Negative Affect, Stress & Self‐efficacy, and Social Relationships. Designed for use across the lifespan (ages 3–85+), it employs self‐report and proxy‐report measures to ensure reliable assessments across different populations and research settings (Salsman et al., 2013). The NIHTB‐EB was selected due to its strong psychometric properties and its ability to capture multidimensional aspects of psychological well‐being (Babakhanyan et al., 2018). Previous studies have employed this tool to assess psychological well‐being in diverse populations, reinforcing its robustness in measuring emotional health. For instance, it has been applied in research comparing well‐being levels between individuals with clinical conditions, such as HIV, and the general population, highlighting its ability to detect meaningful psychological differences (Brody et al., 2023). Additionally, Lopez et al. (2023) utilized the NIHTB‐EB to investigate psychological well‐being in individuals with Parkinson's disease, further supporting its application in clinical research. These studies demonstrate the versatility of the NIHTB‐EB in capturing meaningful variations in psychological well‐being dimensions across different populations, confirming its validity as a robust assessment tool.

The PWB composite score derived from the NIHTB‐EB reflects key psychological dimensions encompassing both hedonic and eudaimonic well‐being. It captures an individual's cognitive evaluation of overall life satisfaction, assessing whether they perceive their life as fulfilling and meaningful. Additionally, it measures positive affect, reflecting the experience of pleasurable emotions such as happiness, joy, excitement, and contentment, which indicate engagement with one's environment. Furthermore, it evaluates meaning and purpose, representing the extent to which individuals feel their lives have direction, significance, and personal growth (Gritters et al., 2025; Salsman et al., 2013).

Once the PWB composite score was used to group individuals into similar PWB levels, we statistically compared a comprehensive array of variables among clusters, including cognitive functions, personality traits, emotional health, and psychiatric and behavioral factors, to uncover meaningful patterns of well‐being.

Summarizing, this study seeks to enhance the understanding of psychological well‐being by identifying naturally occurring well‐being profiles and examining their relationships with emotional, cognitive, social, and personality traits. Based on prior research, we anticipate that individuals with higher well‐being will report greater positive affect, life satisfaction, and social support, while extraversion and neuroticism will serve as key differentiating factors across well‐being clusters (Huppert, 2009). Furthermore, given the established role of cognitive functions in emotional regulation, attention‐related cognitive abilities are expected to contribute to variations in well‐being (Fredrickson & Branigan, 2005). By integrating machine learning techniques, particularly AP clustering, in combination with robust statistical analyses, this study offers a data‐driven approach to understanding the heterogeneity of well‐being. Unlike conventional methods that rely on predefined categories, clustering techniques enable the identification of natural well‐being subgroups, capturing the intricate relationships between psychological factors in a way that traditional methodologies often overlook. These insights have broad implications for both theoretical models of well‐being and practical applications, particularly in the development of personalized mental health interventions aimed at promoting resilience, enhancing social support, and addressing disparities in well‐being.

## MATERIALS AND METHODS

### Participants

The data for this study were obtained from the publicly available and restricted S1200 datasets of the Human Connectome Project (HCP) (Van Essen et al., 2013). The HCP, a collaborative project led by Washington University, the University of Minnesota, and Oxford University, aims to map human brain networks and their behavioral correlates. This initiative involves a large cohort of healthy individuals, examined using advanced imaging technologies and analytical strategies, integrating methods like MRI and DTI with computational techniques to explore brain connectivity, enhance data collection, create innovative tools, and provide comprehensive brain imaging and behavioral data. Participants were primarily recruited from Missouri and surrounding areas, including a large number of twins and non‐twin siblings, using state health records. The sample was designed to broadly reflect the U.S. population while excluding individuals with major psychiatric, neurological, or systemic medical conditions (e.g., schizophrenia, autism, diabetes, hypertension). For a complete description of inclusion and exclusion criteria, please refer to Van Essen et al. (2013). Data files (csv) were downloaded on the 11th of December 2023 and included demographic data, behavioral scores, and emotional health scores. To reduce potential confounds in the assessment of psychological well‐being, participants with a history of alcohol or drug abuse were removed from the analysis. The initial sample consisted of 1,206 individuals; after filtering substance use, 756 participants remained. Subsequent removal of participants with missing data across relevant variables yielded a final sample of 685 individuals, 313 males and 372 females (Descriptive Statistics in Table 1). KNIME 4.6.1 (Sarica et al., 2014) was used to filter and merge HCP tables and to remove rows with missing data. No imputation techniques were applied. Given prior evidence that subjective well‐being can vary across racial and ethnic groups (Wadsworth & Pendergast, 2021), only White participants ‐the largest homogeneous subgroup‐ were included in this study to avoid confounding effects related to racial heterogeneity.

**TABLE 1:** Descriptive Statistics and Frequencies of the HCP Young Adult Cohort.

	Mean (or frequencies)	Median	SD	IQR	Min	Max
Male/Female	313/372	‐	‐	‐	‐	‐
Age	29.048	29	3.543	6.00	22	36
Education (in years)	15.203	16	1.640	2.00	11	17
Single/In a Relationship	320/365	‐	‐	‐	‐	‐
MMSE	29.089	29	0.962	1.00	23	30
Cognition Total Composite Score	123.999	123	13.506	19.96	91.0	153
PWB	53.043	52.9	7.539	9.50	26.7	75.5

*Note*: Mean/SD and median/IQR are reported to provide a comprehensive view of the data distribution, considering potential skewness and non‐normality.

Abbreviations: PWB, Psychological Well‐being; MMSE, Mini‐Mental State Examination; SD, Standard Deviation; IQR, Interquartile Range; Min, Minimum; Max, Maximum.

### Measures

A range of validated instruments and methodologies was utilized to comprehensively evaluate participants' psychological, cognitive, and behavioral characteristics, as derived from the Human Connectome Project (HCP) dataset, as described in the following sections.

#### Psychological well‐being (PWB) score

Psychological well‐being (PWB) was assessed using the National Institute of Health Toolbox Emotion Battery (NIHTB‐EB) (Gershon et al., 2010), a validated instrument designed to evaluate emotional health across four primary subdomains: Negative Affect, Psychological Well‐Being, Stress and Self‐Efficacy, and Social Relationships. The NIHTB‐EB is a well‐established tool with documented reliability and validity across large samples (Gershon et al., 2010; Salsman et al., 2013).

Each subdomain captures distinct conceptual dimensions:

- Negative Affect measures three core negative emotions—anger, fear, and sadness—to assess emotional distress.
- Social Relationships evaluates perceived social support, loneliness, and social distress, emphasizing interpersonal well‐being.
- Stress & Self‐Efficacy assesses perceived stress, defined as the extent to which life is experienced as unpredictable or overwhelming, alongside self‐efficacy, or the belief in one's ability to effectively manage challenges.
- Psychological Well‐Being focuses on life satisfaction, positive affect, and meaning and purpose, aligning with hedonic (affective experience) and eudaimonic (cognitive appraisal) dimensions of well‐being (Deci & Ryan, 2008; Ryff et al., 1995).

The PWB composite T‐scores used in this study were derived directly from the HCP dataset and follow the standardization procedures described in Salsman et al. (2013).

For this study, the Psychological Well‐Being composite score was derived from the Life Satisfaction, Positive Affect, and Meaning & Purpose subdomains, following the methodology proposed by Babakhanyan et al. (2018). These subdomains were standardized as T‐scores (mean = 50, SD = 10) to ensure comparability across participants. The Babakhanyan formula integrates these scores into a single global PWB value, offering a multidimensional measure of well‐being in which higher scores indicate greater psychological well‐being. In this study, the PWB composite score was the primary target variable used for clustering participants into distinct groups and examining its associations with cognitive, emotional, and personality variables.

The other NIHTB‐EB subdomains ‐ Negative Affect, Social Relationships, and Stress & Self‐Efficacy ‐ were analyzed separately to explore broader emotional and social factors, ensuring a more comprehensive assessment of well‐being‐related factors.

Given the secondary nature of the data, no additional psychometric analyses were conducted within this study.

#### Cognitive and personality variables

To gain a comprehensive understanding of participants' cognitive, emotional, and personality profiles, cognitive functioning was assessed using the NIH Toolbox Cognition Battery (NIHTB‐CB) (Weintraub et al., 2013). This battery evaluates both fluid and crystallized cognitive abilities through standardized tasks, including the Picture Sequence Memory Test, Dimensional Change Card Sort Test, and Picture Vocabulary Test. From these assessments, composite scores for fluid, crystallized, and total cognition were derived, providing a holistic measure of participants' cognitive capacities (https://nihtoolbox.org/domain/cognition/).

Impulsivity and self‐control were examined using Delay Discounting tasks, where participants chose between smaller immediate rewards and larger delayed rewards. These decisions provided insights into participants' impulsivity and self‐regulation levels (da Matta et al., 2012) (Halcomb, 2023; Myerson et al., 2001).

Personality traits were assessed using the NEO Five‐Factor Inventory (NEO‐FFI) (McCrae & Costa, 2004), a widely used instrument designed to measure five core personality dimensions: Neuroticism, Extraversion, Openness to Experience, Agreeableness, and Conscientiousness.

Further details on specific measures and test descriptions can be found in Section A of the supplementary material.

#### Psychiatric and behavioral measures

The psychiatric and behavioral profiles of participants were assessed using validated psychological tools to capture a broad spectrum of mental health and life functioning indicators.

The Adult Self‐Report (ASR) (Guerrero et al., 2020) was used to evaluate Syndrome Scales, which measure key psychological dimensions, including Anxious/Depressed, Withdrawn, Somatic Complaints, Thought Problems, Aggressive Behavior, Rule‐Breaking Behavior, and Intrusive Behaviors. Additionally, DSM‐Oriented Scales were analyzed, focusing on Depressive Problems, Anxiety Problems, Somatic Problems, Avoidant Personality Problems, and Antisocial Personality Problems.

To further explore behavioral and psychiatric history, the Semi‐Structured Assessment for the Genetics of Alcoholism (SSAGA) (Bucholz et al., 1994) was utilized. This comprehensive diagnostic interview, based on DSM criteria, assesses various psychiatric conditions. In this study, Childhood Conduct Problems and Major Depressive Episodes were specifically examined due to their relevance in understanding the relationship between psychological well‐being and behavioral patterns.

These measures provided critical insights into participants' psychiatric and behavioral dimensions, facilitating a nuanced understanding of how these factors intersect with psychological well‐being.

### Affinity Propagation Clustering

To analyze psychological well‐being (PWB) scores and uncover meaningful patterns in the data, we employed Affinity Propagation clustering, a powerful unsupervised learning technique. Clustering is widely used in machine learning to group data points into subsets (clusters) based on their similarity, revealing hidden structures without requiring predefined labels (Jain et al., 1999). This data‐driven approach is particularly valuable for psychological research, where well‐being profiles may emerge naturally rather than being dictated by predefined categories.

Several clustering methodologies exist, each with distinct strengths and limitations. Hierarchical clustering constructs nested groupings by iteratively merging or splitting data points, offering a visual representation via dendrograms. However, this method can be computationally demanding, particularly for large datasets (Jain et al., 1999). Partition‐based methods, such as k‐means clustering, segment data into a fixed number of clusters based on distance metrics. While widely used, k‐means requires an a priori specification of the number of clusters, making it less adaptable to complex psychological datasets, where the optimal number of clusters is often unknown (Brusco et al., 2019).

In contrast, exemplar‐based methods, such as AP, offer a more flexible and adaptive solution. Originally introduced by Frey and Dueck (2007), AP is a non‐hierarchical clustering algorithm that identifies representative data points, or exemplars, which serve as cluster centers. Unlike traditional methods, AP does not require a predefined number of clusters. Instead, it dynamically determines the optimal number of clusters through an iterative message‐passing process between data points (Sarica et al., 2021). This feature makes AP particularly well‐suited for psychological and neurocognitive research, where the underlying group structure is often unknown or heterogeneous (Brusco et al., 2019; Sarica et al., 2021).

It should be noted, however, that while AP eliminates the need to prespecify the number of clusters, it still requires the definition of a preference parameter, which controls how likely each data point is to be chosen as an exemplar. In practice, this shifts the model selection problem from choosing the number of clusters (as in k‐means) to selecting an appropriate preference value. Following Frey and Dueck's (2007) recommendation, we set the preference to the minimum of the similarity values, a common strategy that generally yields a conservative number of clusters. Although this choice is data‐informed, it remains somewhat arbitrary, reinforcing that AP, like all clustering methods, involves tuning decisions that influence final results.

Moreover, AP is conceptually related to the k‐medoids algorithm (also known as the p‐median or k‐median problem), as both are exemplar‐based techniques that aim to optimize intra‐cluster similarity. However, unlike exact optimization algorithms, AP functions as a heuristic: it seeks high‐quality solutions efficiently, but it is not guaranteed to identify the global optimum. Prior research has shown that, when the number of clusters is fixed, k‐medoids often produces better optimization outcomes (i.e., higher objective function values) than AP (Brusco & Köhn, 2008, 2009; Köhn et al., 2010). Therefore, although AP offers computational efficiency and model flexibility, we recognize its limitations as a heuristic (Brusco & Steinley, 2015).

In this study, AP clustering was applied to a single variable, the composite PWB score, to identify distinct well‐being profiles. Clustering on a single variable, in many cases, can be solved optimally using dynamic programming (Fisher, 1958; Steinley & Hubert, 2008). However, we opted for AP in this context because of its flexibility in detecting exemplar‐based structures and its usefulness in psychological applications where even one‐dimensional constructs can exhibit nonlinear distributional patterns.

The algorithm was implemented using Python's (v. 3.8.6) scikit‐learn library (v. 0.23) with key hyperparameters carefully optimized to ensure stability and interpretability of the results. Specifically:

- Clustering Variable: A single composite score for Psychological Well‐Being was used as the basis for similarity computation.
- Similarity Measure: We employed the negative squared Euclidean distance, a standard metric that enhances cluster compactness and separation.
- Preference Value: The preference parameter, which influences the number of clusters, was set as the minimum of the similarity measures (Frey & Dueck, 2007).
- Damping Factor: The damping factor was set to the recommended value of 0.5 to prevent oscillations and ensure the algorithm's convergence to stable clusters (Brusco et al., 2019).

To evaluate the internal validity of the clustering solution, we calculated the silhouette coefficient, a widely used metric that assesses how well each observation fits within its assigned cluster compared to other clusters—balancing intra‐cluster cohesion and inter‐cluster separation. Silhouette scores range from −1 to +1, where values near +1 indicate well‐separated, compact clusters, values around 0 suggest overlapping clusters or ambiguous assignments, and values below 0 indicate possible misclassification. A silhouette score above 0.5 is generally considered indicative of a reasonably well‐structured clustering solution.

### Statistical Analysis

To evaluate whether the clusters identified through AP differed significantly in terms of cognitive, emotional, and personality variables, we employed a non‐parametric ANOVA approach using Jamovi (Version 2.3). Specifically, the Kruskal‐Wallis test was chosen due to its suitability for data that violate the assumptions of normality and homogeneity of variances (Kruskal & Wallis, 1952). These conditions are frequently encountered in psychological datasets, where variables often exhibit skewed or heterogeneous distributions (Micceri, 1989).

The Kruskal‐Wallis test assesses whether median ranks differ significantly across clusters, providing a robust alternative to parametric ANOVA. In this study, statistical significance was set at p < 0.05, and effect sizes were calculated using epsilon‐squared (ε^2^), a metric that quantifies the magnitude of observed differences, offering additional interpretative value (Tomczak & Tomczak, 2014).

To further examine inter‐cluster differences while controlling for covariates such as age and gender, we employed permutation testing. Permutation methods, as described by Freedman and Lane (Freedman & Lane, 1983), provide a flexible and robust framework for hypothesis testing, particularly when traditional parametric methods (e.g., ANCOVA) are unsuitable due to violations of underlying statistical assumptions (Ottoboni et al., 2018). By repeatedly reshuffling the dataset to generate an empirical null distribution, permutation testing enhances the robustness and reliability of statistical comparisons.

For this study, permutation tests were conducted using the “permuco” package (Frossard & Renaud, 2021) in R software (Version 2024.09.1 + 394), enabling precise covariate control while maintaining statistical rigor. The Freedman and Lane permutation method was directly applied to ensure valid hypothesis testing.

Following significant findings, post‐hoc analyses were conducted to determine specific inter‐cluster differences with greater precision. To mitigate the risk of Type I errors due to multiple comparisons, Holm's correction was applied (Giacalone et al., 2018). This method provides a more statistically powerful alternative to the Bonferroni correction, effectively controlling the family‐wise error rate while preserving sensitivity (Holm, 1979).

These statistical analyses allowed us to test our hypotheses regarding inter‐cluster differences in cognitive, emotional, and personality variables, providing deeper insights into the distinct psychological well‐being profiles identified in this study.

## RESULTS

### Cohort characteristics

Descriptive statistics for the HCP Young Adult Cohort are presented in Table 1. The dataset includes 685 participants (313 males, 372 females), with a mean age of 29.05 years (SD = 3.54, range: 22–36). Educational attainment ranged from 11 to 17 years, with a median of 16 years (M = 15.20, SD = 1.64). The cohort was fairly balanced in terms of relationship status, with 320 participants single and 365 in a relationship.

Cognitive functioning was assessed using the Mini‐Mental State Examination (MMSE) and the NIH Toolbox Cognition Battery. Participants demonstrated high cognitive ability, with an average MMSE score of 29.09 (SD = 0.96, range: 23–30). The Cognition Total Composite Score had a mean of 124.00 (SD = 13.51, range: 91.0–153).

Psychological well‐being (PWB) scores, derived from the NIH Toolbox Emotion Battery, had a mean of 53.04 (SD = 7.54, range: 26.7–75.5). Given potential non‐normality in psychological measures, both mean/SD and median/IQR are reported to provide a comprehensive view of the data distribution.

### Cluster Identification through Affinity Propagation

Affinity Propagation clustering, applied to participants' Psychological Well‐Being (PWB) scores, identified four distinct clusters: Low, Medium‐low, Medium‐high, and High. These clusters represent varying levels of well‐being and capture the heterogeneity within the HCP Young Adult cohort. To assess the quality of the clustering solution, we computed the silhouette score, which yielded a value of 0.537, indicating a reasonably well‐structured clustering solution with good internal cohesion and separation between clusters. Table 2 provides descriptive statistics for demographic, cognitive, and psychological variables across the identified clusters.

**TABLE 2:** Descriptive Statistics and Frequencies of the HCP Young Adult Cohort divided by cluster.

	PWB Cluster	Mean (or frequencies)	Median	SD	IQR	Min	Max
**Male/Female**	Low	63/75	‐	‐	‐	‐	‐
	Medium‐low	129/123	‐	‐	‐	‐	‐
	Medium‐high	81/118	‐	‐	‐	‐	‐
	High	40/63	‐	‐	‐	‐	‐
**Age**	Low	29.094	29	3.443	5	22	35
	Medium‐low	29.028	29	3.570	6	22	36
	Medium‐high	29.095	29	3.500	6	22	36
	High	28.938	29.00	3.750	5.25	22	35
**Education (in years)**	Low	14.928	16.00	1.778	2	11	17
	Medium‐low	15.325	16.00	1.573	2	11	17
	Medium‐high	15.342	16	1.542	1.50	11	17
	High	14.990	16.00	1.750	2	11	17
**Single/In a relationship**	Low	91/47	‐	‐	‐	‐	‐
	Medium‐low	125/127	‐	‐	‐	‐	‐
	Medium‐high	76/123	‐	‐	‐	‐	‐
	High	28/68	‐	‐	‐	‐	‐
**MMSE**	Low	29.1	29	0.951	1	26	30
	Medium‐low	29.2	29	0.866	1	26	30
	Medium‐high	29.1	29	1.06	1	23	30
	High	29	29	1.02	2	27	30
**Composite Cognitive Score**	Low	124	123	13.8	21.22	91	153
	Medium‐low	124	123	13.2	19.68	95.9	153
	Medium‐high	125	123	13.8	19.19	96.6	153
	High	123	119	13.3	17.82	92.9	153
**PWB**	Low	42.5	43.6	3.7	5.20	26.7	46.8
	Medium Low	50.9	51.1	1.96	2.96	46.9	54.1
	Medium High	57.2	57.1	1.91	3.12	54.1	61.1
	High	65.2	64.3	3.16	4.61	61.2	75.5

Abbreviations: PWB, Psychological Well‐being; MMSE, Mini‐Mental State Examination; SD, Standard Deviation; IQR, Interquartile Range; Min, Minimum; Max, Maximum.

The distribution of gender varied across clusters, with the Low PWB group comprising 63 males and 75 females, while the High PWB group included 40 males and 63 females. Age distributions were similar across clusters, with mean ages ranging from 28.94 to 29.09 years and no significant differences in age variability.

Regarding education, the median number of years of education was 16 across all clusters, although the Low PWB cluster had a slightly lower mean (14.93 years) compared to the Medium‐high PWB group (15.34 years).

Relationship status showed notable variation, with a higher proportion of single individuals in the Low PWB cluster (91 single, 47 in a relationship) compared to the High PWB group (28 single, 68 in a relationship).

Cognitive performance, assessed using the Mini‐Mental State Examination (MMSE) and the NIH Toolbox Composite Cognitive Score, remained relatively consistent across clusters. The MMSE mean scores ranged between 29.1 and 29.2, with minimal variation. Similarly, Cognition Composite Scores showed no substantial differences, with mean values ranging between 123 and 125 across all clusters.

PWB scores followed a progressive pattern across clusters, with the Low PWB group exhibiting the lowest mean score (42.5) and the High PWB group the highest (65.2). The Medium‐low and Medium‐high clusters showed intermediate values, with means of 50.9 and 57.2, respectively. These results reflect the gradual variation in psychological well‐being within the sample, highlighting significant differences between extreme groups.

To explore and illustrate the relationships between PWB levels and associated variables, radar plots were used to visualize key personality traits across clusters. These plots provide an intuitive representation of how different personality dimensions vary among individuals with different levels of psychological well‐being, offering insights into the psychological and emotional factors linked to well‐being.

**FIGURE 1:**
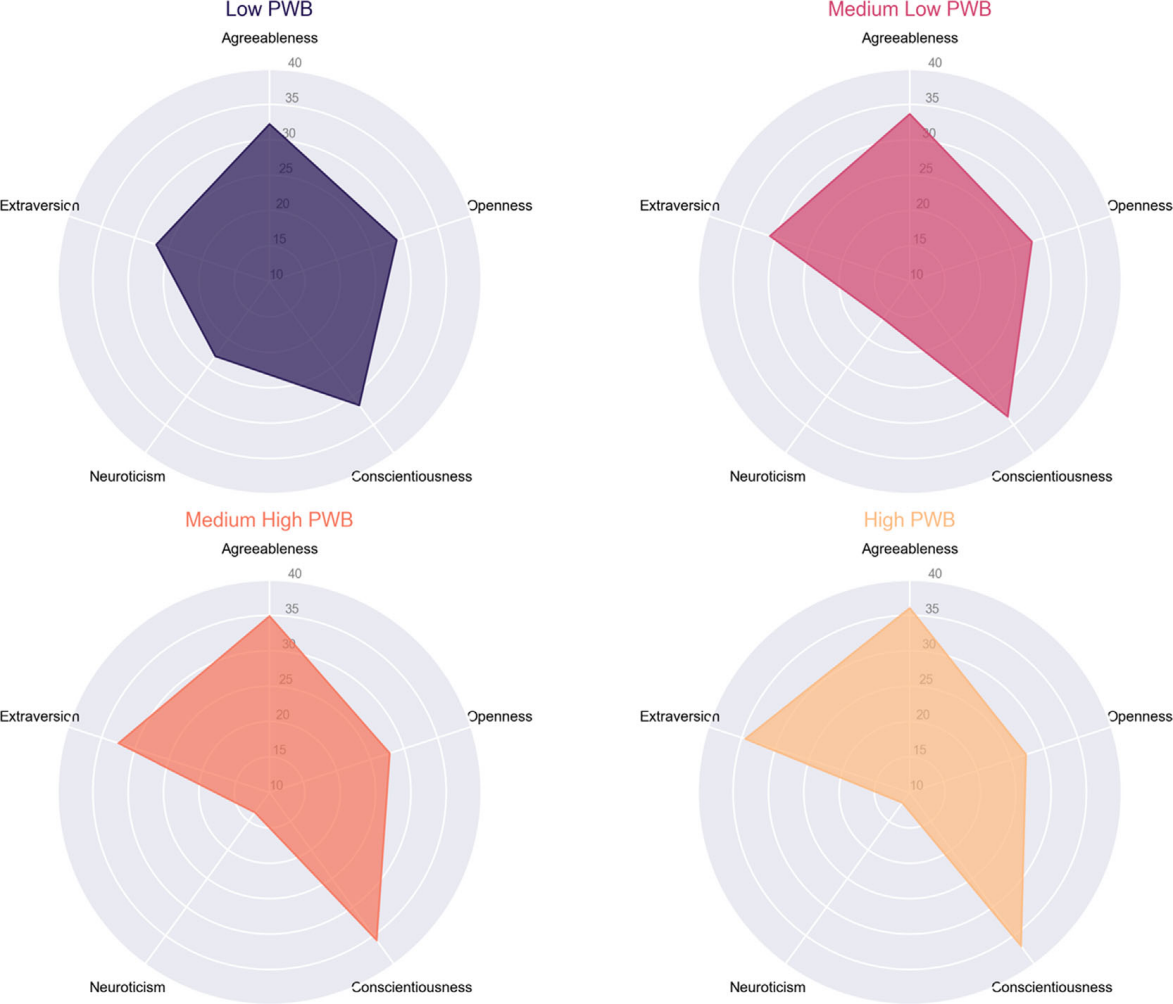
Personality trait profiles across Psychological Well‐Being (PWB) clusters. Radar plots illustrate the distribution of Big Five personality traits (Agreeableness, Extraversion, Openness, Conscientiousness, and Neuroticism) across the four PWB clusters (Low, Medium‐low, Medium‐high, and High PWB). Individuals in the Low PWB cluster exhibit higher neuroticism and lower extraversion, agreeableness, and conscientiousness, whereas higher PWB clusters show increased levels of extraversion, agreeableness, and conscientiousness, suggesting a strong relationship between positive personality traits and well‐being. The plots provide a visual representation of personality differences across well‐being levels, reinforcing the role of emotional stability and social engagement in psychological well‐being.

Figure 1 depicts the Big Five personality traits (Agreeableness, Extraversion, Openness, Conscientiousness, and Neuroticism) across the four PWB clusters (Low, Medium‐low, Medium‐high, and High PWB). The Low PWB cluster exhibits higher neuroticism and lower levels of extraversion, agreeableness, and conscientiousness, suggesting a personality profile characterized by emotional instability and reduced social engagement. In contrast, higher PWB clusters display greater extraversion, agreeableness, and conscientiousness, reinforcing the idea that positive social and emotional traits are strongly associated with greater well‐being.

**FIGURE 2:**
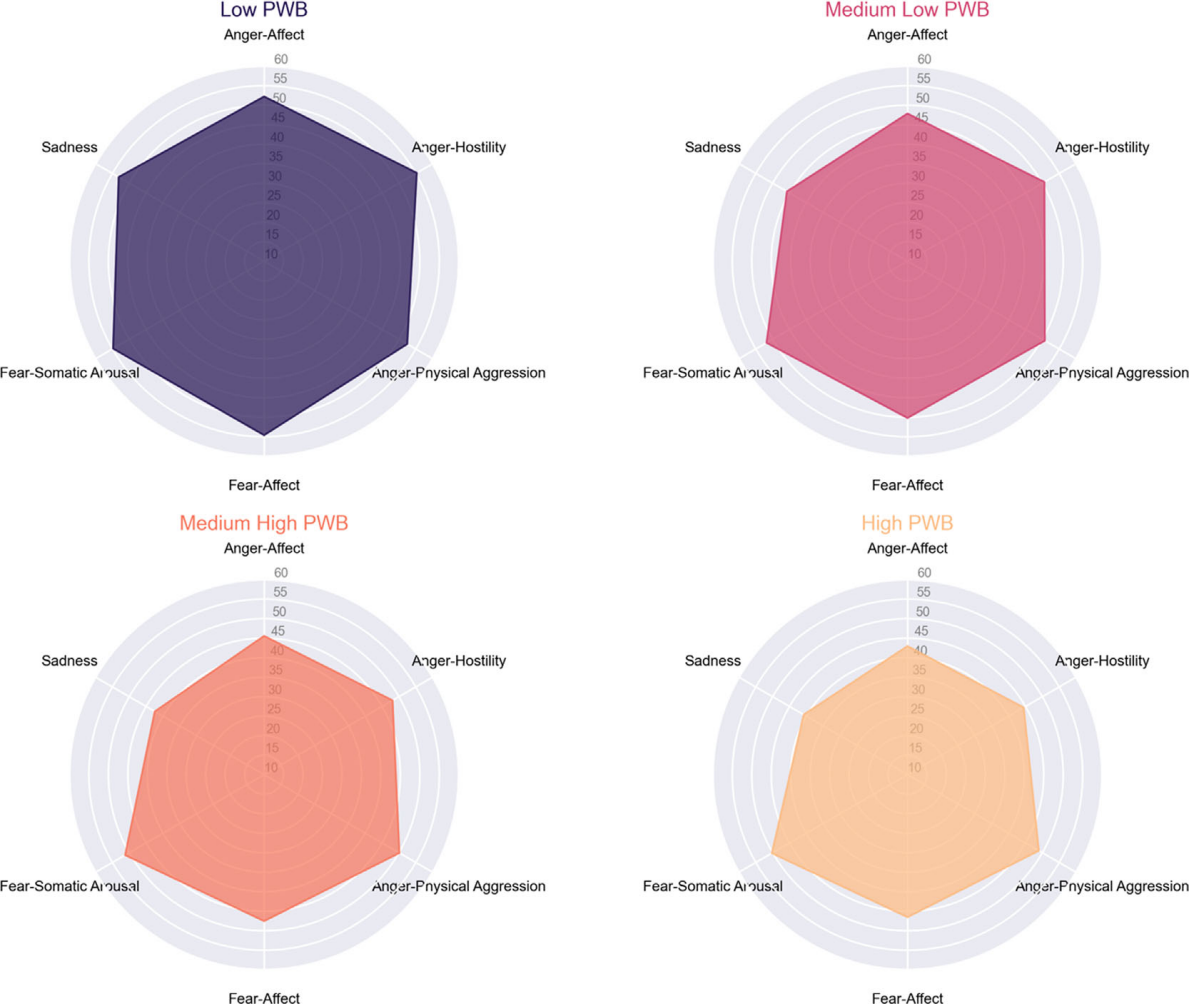
Negative Affect subdomains across Psychological Well‐Being (PWB) clusters. Radar plots depict the distribution of Negative Affect subdomains (Anger‐Affect, Anger‐Hostility, Anger‐Physical Aggression, Fear‐Affect, Fear‐Somatic Arousal, and Sadness) across the four PWB clusters (Low, Medium‐low, Medium‐high, and High PWB). The Low PWB cluster shows the highest levels of anger, sadness, and fear‐related responses, while these negative emotional states progressively decline as PWB increases. Higher PWB clusters exhibit lower distress levels, particularly in anger and sadness, reflecting the strong relationship between emotional regulation and well‐being. These visualizations highlight the role of negative affect in differentiating well‐being profiles, reinforcing its impact on psychological health.

Figure 2 illustrates the Negative Affect subdomains across the four Psychological Well‐Being (PWB) clusters (Low, Medium‐low, Medium‐high, and High PWB), providing insights into the role of emotional distress in differentiating well‐being profiles. The Low and Medium‐low PWB clusters exhibit higher levels of negative emotional states, including Anger‐Affect, Anger‐Hostility, Anger‐Physical Aggression, Fear‐Affect, Fear‐Somatic Arousal, and Sadness. This pattern suggests that individuals with lower psychological well‐being experience greater emotional distress, heightened anger responses, and increased fear‐related symptoms.

In contrast, the Medium‐high and High PWB clusters display lower levels of negative affect, indicating better emotional regulation and resilience. The progressive decrease in anger, sadness, and fear responses across increasing well‐being levels highlights the central role of emotional stability in psychological well‐being. This gradient reinforces previous findings that high psychological well‐being is associated with reduced emotional distress and greater capacity for managing negative emotions.

**FIGURE 3:**
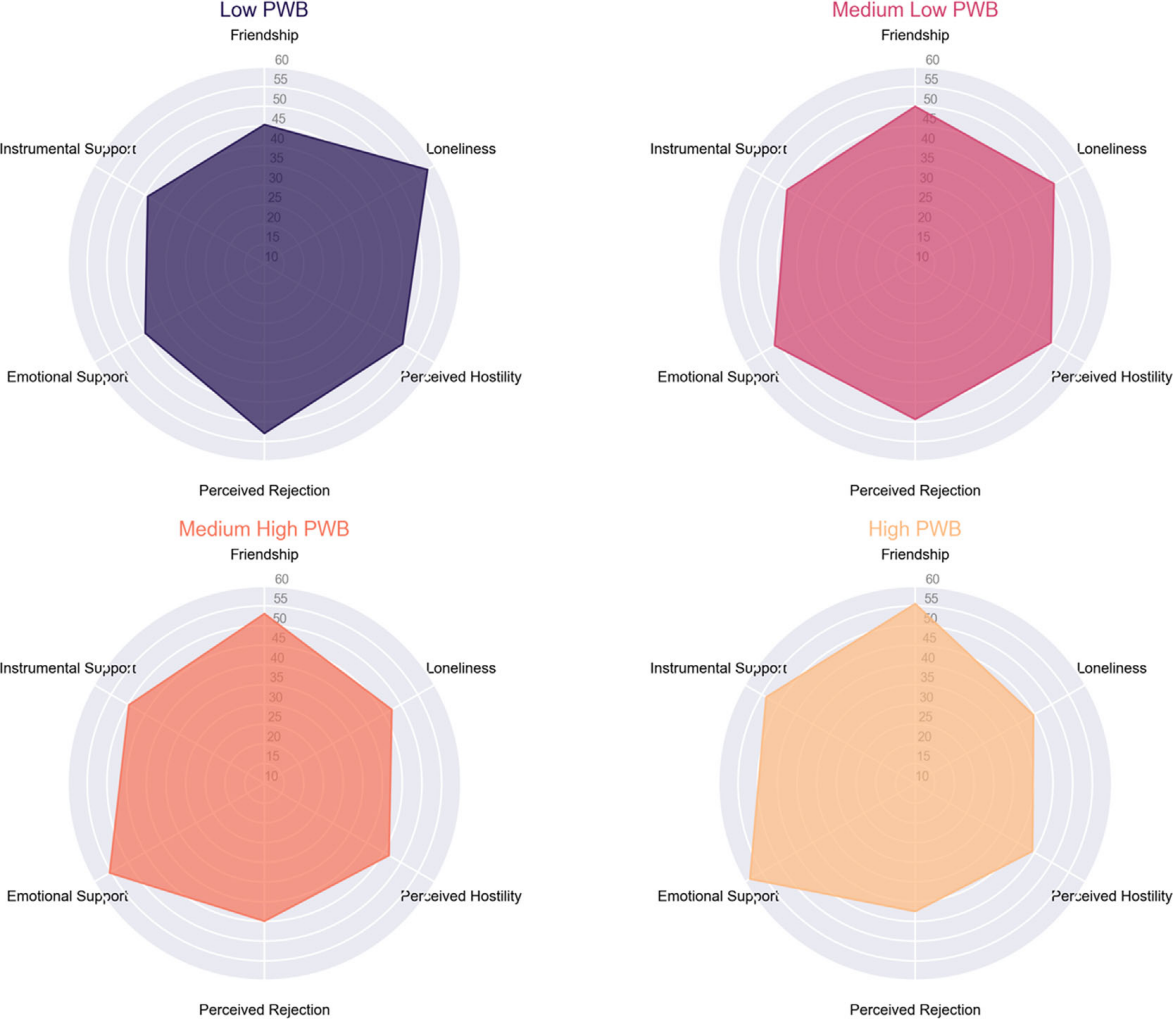
Social Relationships subdomains across Psychological Well‐Being (PWB) clusters. Radar plots illustrate the distribution of Social Relationships subdomains (Friendship, Instrumental Support, Emotional Support, Loneliness, Perceived Rejection, and Perceived Hostility) across the four PWB clusters (Low, Medium‐low, Medium‐high, and High PWB). The Low and Medium‐low PWB clusters exhibit higher levels of loneliness, perceived rejection, and perceived hostility, indicating greater interpersonal challenges. In contrast, the Medium‐high and High PWB clusters show higher levels of friendship, instrumental support, and emotional support, reflecting stronger social connections and support networks. These patterns highlight the critical role of social relationships in well‐being, emphasizing that greater social support is a key factor in higher psychological well‐being levels.

Figure 3 visualizes the Social Relationships subdomains across Psychological Well‐Being (PWB) clusters, providing insights into the association between social factors and well‐being levels. The Low and Medium‐low PWB clusters show greater loneliness, perceived rejection, and perceived hostility, suggesting that individuals with lower well‐being may experience more social isolation and interpersonal distress. These patterns indicate that negative social experiences are strongly linked to lower psychological well‐being, reinforcing previous findings that social disconnection is a risk factor for mental health challenges.

Conversely, the Medium‐high and High PWB clusters exhibit higher levels of friendship, instrumental support, and emotional support, indicating that individuals with greater well‐being tend to have stronger social connections and access to supportive networks. These findings align with the literature on social support as a protective factor for mental health, demonstrating that individuals with higher psychological well‐being are more likely to experience positive social interactions, emotional reinforcement, and practical assistance from their networks.

### Statistical analysis

#### Non‐Parametric Analysis of Variance

A non‐parametric ANOVA was performed to confirm the visual trends observed in the radar plots and ensure statistical robustness. The Kruskal‐Wallis' test revealed significant differences across clusters for several psychological, emotional, and personality variables aligning with the patterns observed in the radar plots. Table 3 provides a comprehensive summary of the ANOVA results, detailing chi‐squared values, degrees of freedom, p‐values, and effect sizes (ε^2^) for all variables analyzed.

**TABLE 3:** Results of the Kruskal‐Wallis Non‐Parametric ANOVA Across Clusters.

Variable	χ^2^	p‐value	ε^2^
MMSE	0.943	0.815	0.001
PSQI	19.324	**< .001**	0.028
Picture Sequence Memory Test	3.452	0.327	0.005
Dimensional Change Card Sort Test	3.596	0.309	0.005
Flanker Inhibitory Control and Attention Test	0.339	0.953	0.000
Oral Reading Recognition Test	5.797	0.122	0.008
Picture Vocabulary Test	1.591	0.661	0.002
Pattern Comparison Processing Speed Test	1.127	0.771	0.002
Delay Discounting_200	0.689	0.876	0.001
Delay Discounting_40K	2.284	0.516	0.003
List Sorting Working Memory Test	4.942	0.176	0.007
Cognition Fluid Composite	0.283	0.963	0.000
Cognition Composite Score	1.176	0.759	0.002
Cognition Crystallized Composite	3.642	0.303	0.005
ER40_Anger	2.581	0.461	0.004
ER40_Fear	2.019	0.568	0.003
ER40_Happiness	0.739	0.864	0.001
ER40_Neutral	1.244	0.743	0.002
ER40_Sadness	2.849	0.416	0.004
Anger‐Affect	102.623	**< .001**	0.150
Anger‐Hostility	114.337	**< .001**	0.167
Anger‐ Physical Aggression	13.621	**0.003**	0.020
Fear‐Affect	87.525	**< .001**	0.128
Fear‐Somatic Arousal	21.177	**< .001**	0.031
Sadness	204.757	**< .001**	0.299
Friendship	89.628	**< .001**	0.131
Loneliness	188.271	**< .001**	0.275
Perceived Hostility	48.546	**< .001**	0.071
Perceived Rejection	123.239	**< .001**	0.180
Emotional Support	154.251	**< .001**	0.226
Instrumental Support	72.645	**< .001**	0.106
Perceived Stress	173.544	**< .001**	0.254
Self‐Efficacy	92.802	**< .001**	0.136
Agreebleness	35.215	**< .001**	0.051
Openness to experience	3.773	0.287	0.006
Conscientiousness	66.426	**< .001**	0.097
Neuroticism	171.424	**< .001**	0.251
Extraversion	108.109	**< .001**	0.158
Age	0.077	0.994	0.000
Handedness	4.516	0.211	0.007
Employment Status	2.542	0.468	0.004
Education (in years)	6.327	0.097	0.009
Relationship Status	38.967	**< .001**	0.057
Anxious/Depressed	189.276	**< .001**	0.277
Withdrawn	117.495	**< .001**	0.172
Somatic Complaints	52.186	**< .001**	0.076
Thought Problems	44.179	**< .001**	0.065
Aggressive Behavior	60.937	**< .001**	0.089
Rule Breaking Behavior	33.262	**< .001**	0.049
Intrusive	7.889	**0.048**	0.012
Depressive Problems	127.056	**< .001**	0.186
Anxiety Problems	92.187	**< .001**	0.135
Somatic Problems	27.865	**< .001**	0.041
Avoidant Personality Problems	117.897	**< .001**	0.172
Antisocial Personality Problems	34.087	**< .001**	0.050
Childhood Conduct Problems	3.013	0.390	0.004
Major Depressive Episode	26.710	**< .001**	0.039

*Note:* Variables with a significant p‐value (p < .05) are highlighted in bold.

Abbreviations: df, degrees of freedom; ER, Emotion Recognition; MMSE, Mini‐Mental State Examination; PSQI, Pittsburgh Sleep Quality Index.

Cognitive measures from the NIH Toolbox Cognition Battery (NIHTB‐CB), such as the Composite Cognitive Score (χ^2^ = 1.176, p = 0.759, ε^2^ = 0.002) and specific tasks like the Picture Sequence Memory Test (χ^2^ = 3.452, p = 0.327, ε^2^ = 0.005), did not show significant variation across clusters. Similarly, emotion recognition tasks, including ER40_Anger, ER40_Fear, and ER40_Happiness, yielded no significant differences across groups (all p > 0.05). These findings suggest that cognitive and emotional recognition measures may be less closely linked to psychological well‐being clusters.

In contrast, variables from the NIH Toolbox Emotion Battery demonstrated consistent and robust differences between clusters. Notable examples include Anger‐Affect (χ^2^ = 102.623, p < 0.001, ε^2^ = 0.150), Sadness (χ^2^ = 204.757, p < 0.001, ε^2^ = 0.299), and Perceived Stress (χ^2^ = 173.544, p < 0.001, ε^2^ = 0.254). These variables exhibited some of the highest effect sizes, underscoring their strong differentiation between clusters and highlighting the central role of emotional dimensions in psychological well‐being.

Personality traits also showed significant differences across clusters. Neuroticism (χ^2^ = 171.424, p < 0.001, ε^2^ = 0.251) and Extraversion (χ^2^ = 108.109, p < 0.001, ε^2^ = 0.158)varied markedly, while Agreeableness (χ^2^ = 35.215, p < 0.001, ε^2^ = 0.051) and Conscientiousness (χ^2^ = 66.426, p < 0.001, ε^2^ = 0.097) progressively increased in clusters with higher psychological well‐being. In contrast, Openness to Experience (χ^2^ = 3.773, p = 0.287, ε^2^ = 0.006) did not show significant variability, as already observed in Figure 1, suggesting its limited role in differentiating well‐being levels.

Psychiatric and behavioral measures showed pervasive differences across clusters. For example, Anxious/Depressed (χ^2^ = 189.276, p < 0.001, ε^2^ = 0.277) and Depressive Problems (χ^2^ = 127.056, p < 0.001, ε^2^ = 0.186) were markedly elevated in the Low cluster, reflecting heightened psychological distress. In contrast, Intrusive Behavior (χ^2^ = 7.889, p = 0.048, ε^2^ = 0.012) was close to the significance threshold, while Childhood Conduct Problems (χ^2^ = 3.013, p = 0.390, ε^2^ = 0.004) were not significant.

Demographic variables presented mixed results. Age (χ^2^ = 0.077, p = 0.994, ε^2^ = 0.000) and Handedness (χ^2^ = 4.516, p = 0.211, ε^2^ = 0.007) showed no significant differences across clusters. Conversely, Relationship Status (χ^2^ = 38.967, p < 0.001, ε^2^ = 0.057) and sleep quality, as assessed by the PSQI (χ^2^ = 19.324, p < 0.001, ε^2^ = 0.028), were significantly associated with cluster membership.

These findings highlight the multifaceted nature of psychological well‐being. Clusters differed most markedly in emotional, behavioral, and personality domains, while demographic and cognitive variables showed more limited associations.

#### 3.3.2 Permutation test and post‐hoc analysis

To further verify the differences between the four clusters, post‐hoc analyses were performed using a permutation‐based approach, focusing exclusively on variables that showed significant differences across clusters in the initial ANOVA, with age and sex included as covariates. Multiple comparisons were corrected using Holm's method. The results are shown in Table 4.

**TABLE 4:** Permutation Tests and Post‐Hoc Comparisons Across Clusters.

Variable	p‐Value	Post‐Hoc
PSQI	<.001	L > Ml ‐ L > Mh ‐ L > H
Anger‐Affect	<.001	L > Ml, L > Mh, L > H
Anger‐Hostility	<.001	L > Ml, L > Mh, L > H, Ml > Mh, Ml > H, Mh > H
Anger‐Physical Aggression	<.001	L > Ml, L > Mh, L > H
Fear‐Affect	<.001	L > Ml, L > Mh, L > H, Ml > Mh, Ml > H
Fear‐Somatic Arousal	<.001	L > Ml, L > Mh, L > H
Sadness	<.001	L > Ml, L > Mh, L > H, Ml > Mh, Ml > H
Friendship	<.001	L < Ml, L < Mh, L < H, Ml < Mh, Ml < H, Mh < H
Loneliness	<.001	L > Ml, L > Mh, L > H, Ml > Mh, Ml > H, Mh > H
Perceived Hostility	<.001	L > Mh, L > H, Ml > Mh, Ml > H
Perceived Rejection	<.001	L > Ml, L > Mh, L > H, Ml > Mh, Ml > H, Mh > H
Emotional Support	<.001	L < Ml, L < Mh, L < H, Ml < Mh, Ml < H, Mh < H
Instrumental Support	<.001	L < Ml, L < Mh, L < H, Ml < Mh, Ml < H, Mh < H
Perceived Stress	<.001	L > Ml, L > Mh, L > H, Ml > Mh, Ml > H, Mh > H
Self‐Efficacy	<.001	L > Ml, L > Mh, L > H, Ml > Mh, Ml > H, Mh > H
Agreeableness	<.001	L < Ml, L < Mh, L < H, Ml < H
Conscientiousness	<.001	L < Ml, L < Mh, L < H, Ml < Mh, Ml < H
Neuroticism	0.034	L > Ml, L > Mh, L > H, Ml > Mh, Ml > H, Mh > H
Extraversion	<.001	L < Ml, L < Mh, L < H, Ml < Mh, Ml < H, Mh < H
Relationship Status	<.001	L‐Ml, L‐Mh, L‐H, Ml‐H
Anxious/Depressed	<.001	L > Ml, L > Mh, L > H, Ml > Mh, Ml > H, Mh > H
Withdrawn	<.001	L > Ml, L > Mh, L > H, Ml > Mh, Ml > H, Mh > H
Somatic Complaints	<.001	L > Ml, L > Mh, L > H, Ml > Mh, Ml > H
Thought Problems	<.001	L > Ml, L > Mh, L > H, Ml > H
Aggressive Behavior	<.001	L > Ml, L > Mh, L > H, Ml > H
Rule Breaking Behavior	<.001	L > Ml, L > Mh, L > H, Ml > H
Intrusive	0.115	‐
Depressive Problems	<.001	L > Ml, L > Mh, L > H, Ml > Mh, Ml > H
Anxiety Problems	<.001	L > Ml, L > Mh, L > H, Ml > Mh, Ml > H
Somatic Problems	<.001	L > Ml, L > Mh, L > H, Ml > Mh, Ml > H
Avoidant Personality Problems	<.001	L > Ml, L > Mh, L > H, Ml > Mh, Ml > H
Antisocial Personality Problems	<.001	L > Ml, L > Mh, L > H, Ml > H
Major Depressive Episode	<.001	L > Ml, L > Mh, L > H

Abbreviations: L, Low; Ml, Medium‐low; Mh, Medium‐high; H, High; < minor; > major.

Permutation testing, controlling for age and gender, revealed significant differences between clusters across multiple emotional and behavioral variables. Post‐hoc analyses with Holm's correction further clarified the specific inter‐cluster differences. Variables from the NIHTB‐EB showed strong differentiation between clusters. For instance, negative variables such as Anger‐Affect, Fear‐Affect, and Sadness consistently displayed higher scores in the low and medium‐low PWB levels than the other clusters. Conversely, positive variables like Friendship and Emotional Support were lowest in the Low and Medium‐low clusters, progressively increasing in the Medium‐high and High clusters (all p < 0.001). These patterns align closely with those observed in the radar plots, which visually highlighted the differences in emotional distress and social support across PWB levels.

Personality traits also highlighted notable differences. Neuroticism was significantly higher in the Low cluster compared to all others, while Agreeableness and Conscientiousness were progressively higher in clusters with High levels of PWB.

Behavioral and psychiatric variables from the Adult Self‐Report (ASR) and SSAGA measures revealed significant differences. For example, Anxious/Depressed, Withdrawn, and Somatic Complaints were markedly elevated in the Low cluster relative to all other groups, reflecting heightened psychological distress. Similarly, the PSQI scores were highest in the Low PWB cluster, indicating poorer sleep quality. While many variables displayed significant inter‐cluster differences, Intrusive Behavior failed to reach significance (p = 0.115), suggesting a lesser role in differentiating between clusters.

The complete descriptive statistics and frequencies of the HCP Young Adult Cohort divided by cluster are reported in Section B of the supplementary material (Tables S1, S2, and S3).

These findings collectively highlight a clear influence of emotional, social, and behavioral functioning across the clusters, underscoring the complex interplay between well‐being, personality, and behavioral dimensions.

## DISCUSSION AND CONCLUSIONS

The present study aimed to explore differences in emotional, cognitive, personality, and behavioral variables across distinct psychological well‐being (PWB) profiles. Using Affinity Propagation clustering, we identified four naturally occurring clusters of PWB: Low, Medium‐low, Medium‐high, and High. By examining inter‐cluster differences, this study offers a deeper understanding of the multifaceted nature of well‐being and its associations with key psychological constructs.

Contrary to expectations, cognitive variables did not significantly differentiate PWB clusters, a finding supported by both non‐parametric ANOVA (Kruskal‐Wallis) and permutation tests. This aligns with research suggesting that the link between cognitive abilities and well‐being is often indirect, mediated by factors such as emotional stability and personality traits (Ali et al., 2013; Jokela, 2022; Yazdani & Siedlecki, 2021). These findings suggest that while cognition is crucial for everyday functioning, its direct impact on subjective well‐being may be limited. However, there may also be alternative explanations for the absence of significant differences. First, the tools employed to assess cognitive functioning (e.g., MMSE and NIHTB‐CB) may not have been sufficiently sensitive to detect subtle inter‐individual differences in a non‐clinical population. Second, the high level of homogeneity in our sample, composed predominantly of healthy, well‐educated young adults, may have reduced the variability in cognitive performance. This restricted range could limit the power to detect meaningful associations between cognition and psychological well‐being. Future research should examine these dynamics in more heterogeneous populations, employ more sensitive cognitive measures, and further investigate the potential moderating role of personality and emotional regulation in this relationship.

### The Role of Emotion in Differentiating Well‐Being Profiles

A strong gradient in negative affect was observed across clusters, with higher levels of Anger‐Affect, Hostility, and Sadness in the Low and Medium‐low PWB clusters. These findings reinforce the critical role of emotional regulation in well‐being, as anger and hostility are linked to heightened stress responses, maladaptive coping, and poor mental health outcomes. Similarly, sadness, a key marker of depressive states, is associated with cognitive distortions (e.g., negative self‐appraisal) and persistent low mood, both of which impair well‐being (Karam et al., 2024). Given their direct emotional valence, these variables primarily reflect the hedonic dimension of well‐being, as they relate to affective experience and emotional discomfort. These findings highlight the importance of targeted interventions, focusing on emotion regulation and resilience‐building to enhance psychological well‐being.

### Perceived Stress, Self‐Efficacy, and Social Connectedness

Beyond emotional factors, perceived stress and self‐efficacy also significantly differed across clusters. Individuals in the Low and Medium‐low PWB clusters reported higher perceived stress and lower self‐efficacy, suggesting that the way individuals appraise stress and their belief in their ability to manage it is critical factors in well‐being stratification. These results align with models of stress and coping, which emphasize that self‐efficacy buffers against psychological distress by enhancing one's ability to manage life challenges effectively. Self‐efficacy, in particular, maps onto the eudaimonic component of well‐being, as it reflects a sense of purpose, agency, and effective functioning. Perceived stress, though more emotional, can also compromise both hedonic pleasure and the pursuit of meaningful engagement.

Social factors also emerged as key differentiators of well‐being profiles. The Low and Medium‐low PWB clusters exhibited higher levels of Loneliness, Perceived Rejection, and Perceived Hostility, reinforcing the idea that negative interpersonal experiences are closely associated with lower psychological well‐being. Conversely, individuals in the Medium‐high and High PWB clusters reported greater levels of Friendship, Emotional Support, and Instrumental Support, emphasizing the protective role of social connectedness in fostering psychological resilience (Chang et al., 2023; Shaheen et al., 2021). These social dynamics contribute to both hedonic (e.g., emotional closeness, social enjoyment) and eudaimonic (e.g., sense of belonging, relational purpose) dimensions of well‐being.

However, it is important to interpret these findings within the context of the social background of the sample. Participants were drawn from the HCP Young Adult cohort, a relatively homogeneous population with higher‐than‐average levels of education and limited racial, ethnic, and socioeconomic diversity. This restricted variability may have influenced the distribution of social functioning measures and potentially attenuated the effects of social adversity. Future research should explore these associations in more diverse and socioeconomically varied samples to fully capture the impact of social background on psychological well‐being.

Additionally, relationship status significantly differed across clusters, with individuals in lower PWB clusters more likely to be single compared to those in Medium‐high and High clusters. This pattern is consistent with prior literature showing that romantic partnerships may serve as a buffer against psychological distress by providing emotional closeness and everyday support (Dush & Amato, 2005). These findings emphasize the need for interventions that foster social inclusion, enhance peer relationships, and provide accessible support networks to improve well‐being outcomes.

### Personality Traits and Their Influence on Well‐Being

Personality traits played a critical role in differentiating well‐being clusters. Neuroticism was the strongest negative predictor, with significantly higher scores in the Low PWB cluster, in line with prior research linking neuroticism to heightened emotional reactivity, lower social support, and increased stress vulnerability (Yu et al., 2024). This trait is closely tied to compromised hedonic functioning, given its association with frequent negative affect. In contrast, Agreeableness, Conscientiousness, and Extraversion exhibited an upward trend across well‐being clusters, reaffirming their protective role in well‐being by fostering positive interpersonal interactions, goal‐directed behaviors, and resilience. Extraversion contributes to hedonic well‐being through sociability and positive emotions, while Conscientiousness supports eudaimonic well‐being by promoting self‐discipline, purpose, and achievement. Interestingly, Openness to Experience did not significantly differ across clusters. This outcome may be attributed to several factors. First, the relatively homogeneous age range of the sample may limit the variability of the traits. Research indicates that Openness tends to increase during adolescence, remain relatively stable in early adulthood, and decline later in life, suggesting that age‐related variability may be limited in this population (McCrae et al., 1999). Second, the short form of the NEO‐FFI used in this study may lack the sensitivity of the full version to detect subtle individual differences in Openness, which could contribute to the null result. Furthermore, prior research suggests that the role of Openness in psychological well‐being is context‐dependent and may become more salient during periods of major life transition, such as entering the workforce or forming a family (Kokko et al., 2013). Therefore, while Openness may contribute to well‐being, its influence might be harder to detect.

### Psychiatric and Behavioral Factors in Well‐Being Profiles

Psychiatric and behavioral measures further underscored the interplay between mental health and well‐being. Individuals in the Low PWB cluster exhibited higher levels of Anxious/Depressed symptoms and Somatic Complaints, reinforcing the well‐established association between psychological distress and diminished well‐being (Santini et al., 2020). Additionally, poor sleep quality, as measured by the PSQI, was a defining feature of the Low PWB cluster, supporting research that links sleep disturbances to emotional dysregulation and lower life satisfaction (Min et al., 2015; Zhai et al., 2018). These results further highlight the importance of addressing mental health concerns and sleep‐related issues as part of comprehensive well‐being interventions.

### Limitations and Future Directions

While this study provides valuable insights into the psychological well‐being profiles of young adults, several limitations must be acknowledged. First, the sample was restricted to individuals aged 22–36 years, which limits the generalizability of the findings to other age groups, particularly adolescents and older adults who may experience different trajectories of psychological well‐being. Future research should explore whether similar well‐being subgroups emerge in broader age ranges and examine how age‐related factors, such as life transitions and cognitive changes, influence these clusters.

Another important limitation concerns the sample composition, as participants were exclusively drawn from the Human Connectome Project (HCP) and predominantly identified as White. This homogeneity constrains the applicability of the findings to more diverse racial, ethnic, and socioeconomic groups. Given that cultural and environmental factors play a crucial role in shaping well‐being, future studies should aim to include more diverse and representative samples to enhance external validity and ensure the generalizability of results across different demographic groups.

The cross‐sectional design of this study also limits its ability to establish causal relationships between psychological well‐being and associated variables. While significant differences were identified between clusters, the temporal dynamics and potential bidirectional influences among emotional, cognitive, personality, and social factors remain unexplored. Longitudinal studies are needed to examine how well‐being patterns evolve over time, how they interact with life events and stressors, and whether specific interventions can alter an individual's placement within these clusters. A longitudinal approach would also provide deeper insights into whether certain well‐being profiles are more stable or more susceptible to change due to external influences such as major life transitions, chronic stress, or psychological interventions.

### Conclusion and Practical Implications

This study contributes to applied psychology and mental health research by identifying distinct psychological well‐being (PWB) profiles and their associations with emotional, social, and personality factors. By employing Affinity Propagation clustering, this approach enabled the unbiased identification of naturally occurring groups, offering a more nuanced understanding of well‐being stratification beyond traditional models. The findings underscore the role of emotional regulation, personality traits, and social connectedness as key differentiators of well‐being, reinforcing the importance of addressing these dimensions in both research and intervention strategies.

Beyond its theoretical contributions, this study has significant practical implications for mental health interventions and personalized well‐being strategies. The identification of distinct PWB clusters provides a framework for developing targeted intervention programs tailored to the specific psychological and social needs of individuals within each group. Individuals in the Low PWB cluster, characterized by high distress and low self‐efficacy, may benefit from interventions focused on emotion regulation, cognitive restructuring, and resilience‐building. Those in moderate well‐being clusters with limited social support could benefit from initiatives aimed at enhancing peer relationships, fostering social engagement, and strengthening support networks. Meanwhile, individuals in the High PWB cluster, who demonstrate strong emotional and social resources, may serve as potential role models in peer support programs, reinforcing positive interpersonal exchanges and community‐based well‐being initiatives.

These findings highlight the importance of integrating personalized mental health strategies into clinical, workplace, and community settings to enhance psychological resilience and reduce disparities in well‐being. Future research should investigate the longitudinal stability of these clusters and examine how well‐being profiles interact with life stressors, transitions, and targeted interventions. Expanding these findings to clinical settings could further optimize mental health strategies and inform the development of evidence‐based programs that foster emotional well‐being, social support, and adaptive coping mechanisms. By advancing our understanding of the natural clustering of well‐being profiles, this study provides a valuable foundation for designing interventions that promote long‐term psychological health and resilience.

## CONFLICT OF INTEREST STATEMENT

The authors declare no conflict of interest.

## ETHICS STATEMENT

The study was conducted using publicly available datasets, and no ethical approval was required according to institutional policies.

## Supporting information


**Table S1.** Complete descriptive Statistics and Frequencies of the HCP Young Adult Cohort divided by cluster
**Table S2.** Participant's employment status
**Table S3.** Number of Childhood Conduct problems

## Data Availability

The data used in this study were obtained from the restricted‐access Human Connectome Project (HCP) Young Adult S1200 dataset. Access to the restricted data requires approval from the HCP Data Use Agreement (https://db.humanconnectome.org). The authors do not have the authority to share the dataset directly.
