# Supplementary material for: Social, emotional, and personality factors shape four psychological well‐being profiles: A clustering approach in young adults with affinity propagation algorithm
Source: Appl Psychol Health Well Being. 2025 Aug 29;17(5):e70072. doi: 10.1111/aphw.70072 (PMC12397719; doi:10.1111/aphw.70072)
Supplement: Supplementary file 1 — Table S1. Complete descriptive Statistics and Frequencies of the HCP Young Adult Cohort divided by cluster Table S2. Participant's employment status Table S3. Number of Childhood Conduct problems [file APHW-17-0-s001.docx]

**Social, Emotional, and Personality Factors Shape Four Psychological Well-Being Profiles: A Clustering Approach in Young Adults with Affinity Propagation Algorithm**

SUPPLEMENTARY MATERIAL

**Section A: Test Descriptions**

MMSE: The Mini-Mental State Examination (MMSE) is a brief test widely used to screen for cognitive impairment and dementia. The original test, developed by Folstein et al. (1975), includes questions assessing orientation, attention, recall, and language. Galasko et al. (1990) developed a shorter version of the test (Modified MMSE), which is equally sensitive as the full version. A score of 23 out of a possible 30 is commonly recommended as the cut-off for dementia (Folstein et al., 1975).

PSQI: The Pittsburgh Sleep Quality Index (PSQI) (Buysse et al., 2006) consists of 19 self-rated questions and five additional questions answered by bedmates or roommates. The self-rated items generate seven component scores (each ranging from 0 to 3): sleep quality, sleep latency, sleep duration, habitual sleep efficiency, sleep disturbances, use of sleeping medication, and daytime dysfunction. The sum of these seven component scores yields a global score of subjective sleep quality (ranging from 0 to 21). Higher scores indicate poorer subjective sleep quality.

The NIH Toolbox Cognition Battery (NIHTB-CB), recommended for ages 7+, consists of tests of multiple cognitive constructs. It yields individual test scores and the following summary scores: Total Cognition Composite, Fluid Composite (includes Dimensional Change Card Sort, Flanker Inhibitory Control and Attention, Picture Sequence Memory, List Sorting Working Memory, and Pattern Comparison tests), and Crystallized Composite (includes Picture Vocabulary and Oral Reading Recognition tests) (<https://nihtoolbox.org/domain/cognition/>).

- Picture Sequence Memory Test (PSMT): It assesses episodic memory for ages 3–85, focusing on the acquisition, storage, and recall of new information. It is considered a strong fluid ability measure, with performance peaking in early adulthood and declining across the lifespan. Participants recall increasingly lengthy sequences of illustrated objects and activities presented in a specific order on a computer screen. Scores are based on the ability to recall adjacent pairs of pictures, with the final score reflecting the cumulative number of correct adjacent pairs across both learning trials. There is no delayed recall component (Loring et al., 2019).
- Dimensional Change Card Sort Test: This test measures executive function, specifically cognitive flexibility, for ages 3–85. It is considered a fluid ability measure, with performance increasing through childhood and declining in adulthood. Participants match a series of bivalent test pictures (e.g., yellow balls and blue trucks) to target pictures based on one dimension (e.g., color) and later switch to another dimension (e.g., shape). Switch trials require participants to change the matching rule, demanding cognitive flexibility. Scores are based on a combination of accuracy and reaction time
- Flanker Inhibitory Control and Attention Test: The Flanker Test measures executive function, specifically inhibitory control and attention, for ages 3–85. This test is a version of the Eriksen flanker task derived from the Attention Network Test (Rueda et al., 2004). It is considered a fluid ability measure, where performance peaks in early adulthood and declines across the lifespan. Participants focus on a central stimulus while ignoring surrounding flanker stimuli (arrows). Stimuli can be congruent (pointing in the same direction) or incongruent (pointing in opposite directions). Scores are based on a combination of accuracy and reaction time (Foy & Foy, 2020).
- Oral Reading Recognition Test: The Reading Test is a CAT (computer-adaptive test) format measure of reading decoding skill and crystallized abilities for ages 7-85. Those abilities are generally more dependent upon past learning experiences and consistent across the lifespan. The participant is asked to read and pronounce letters and words as accurately as possible. Higher scores indicate better reading ability.
- Picture Vocabulary Test: It is a CAT format measure of general vocabulary knowledge for ages 3-85 and is considered to be a strong measure of crystallized abilities. The participant is presented with an audio recording of a word and four photographic images on the computer screen and is asked to select the picture that most closely matches the meaning of the word. Higher scores indicate higher vocabulary ability.
- Pattern Comparison Processing Speed Test: This test measures processing speed, considered a fluid ability. Processing speed improves throughout childhood and adolescence, then begins to decline in adulthood. For ages 7–85, participants quickly determine whether two side-by-side pictures are the same or different. The items are simple, focusing on measuring pure processing speed. Higher scores indicate faster processing speed.
- List Sorting Working Memory Test: This task assesses working memory and requires the participant to sequence different visually and orally presented stimuli (foods and animals) in order of size. It assesses both information processing and storage and is considered a fluid ability measure. Higher scores indicate better working memory (Weintraub et al., 2013).
- Cognition Fluid Composite Score: This score is derived by averaging the normalized scores of each of the Toolbox tests that are fluid ability measures (Flanker, Dimensional Change Card Sort, Picture Sequence Memory, List Sorting, and Pattern Comparison), then deriving scale scores based on this new distribution. One can interpret the Fluid Cognition Composite as a more global assessment of individual and group fluid cognition functioning. Higher scores indicate higher levels of functioning.
- Cognition Crystallized Composite: This score is derived by averaging the normalized scores of each of the Toolbox tests that are crystallized measures (Picture Vocabulary and Reading Tests), then deriving scale scores based on this new distribution. One can interpret the Crystallized Cognition Composite as a more global assessment of individual and group verbal reasoning. Higher scores indicate higher levels of functioning.
- Cognition Total Composite Score: This score is derived by averaging the normalized scores of both fluid and crystallized cognition measures. Scale scores are derived from this distribution, offering an overall assessment of cognitive functioning. Higher scores indicate better cognitive functioning.

Delay Discounting ($200 and $40,000): This is a well-established measure used to evaluate an individual's preference for smaller immediate rewards over larger delayed ones, often interpreted as an indicator of impulsivity and self-control (da Matta et al., 2012). During the task, participants are presented with a series of binary choices between receiving a smaller monetary reward immediately or a larger reward after a specified delay (Halcomb, 2023). The degree to which individuals discount the value of delayed rewards is quantified using the Area Under the Curve (AUC), a widely accepted summary metric. Lower AUC values indicate steeper discounting (a stronger preference for immediate rewards), while higher AUC values reflect a greater willingness to wait for larger delayed rewards (Myerson et al., 2001).

The NIH Toolbox Emotion Battery (NIHTB-EB) is a self-report measure recommended for ages 8+. It includes four major domains: Psychological Well-Being (used as our target variable), Stress and Self-Efficacy, Social Relationships, and Negative Affect.

The Negative Affect subdomain covers three primary negative emotions: anger, fear, and sadness.

The Social Relationship subdomain includes perceptions of social support, loneliness, and social distress.

The Stress and Self-Efficacy subdomain focuses on perceived stress and self-efficacy.

Subdomains and Measures:

- Anger-Affect: Assesses anger as an emotion for ages 18-85, using a CAT format.
- Anger-Hostility: Assesses attitudes of hostility and cynicism through self-report.
- Anger-Physical Aggression: Assesses aggression as a behavioral component.
- Fear-Affect: Assesses fear and anxious misery for ages 18-85, using a CAT format.
- Fear-Somatic Arousal: Assesses somatic symptoms of anxiety reflecting autonomic arousal for ages 18-85, using a 6-item fixed-length form.
- Sadness: Assesses sadness for ages 18-85.
- Friendship: Assesses perceptions of friendship using an 8-item fixed-length form for ages 18-85.
- Loneliness: Assesses perceptions of loneliness using a 5-item fixed-length form for ages 18-85.
- Perceived Hostility: This measure assesses perceptions of hostility in daily social interactions (e.g., how often people argue with me, yell at me, or criticize me) using an 8-item fixed-length form for people ages 18-85.
- Perceived Rejection: This measure assesses perceptions of social rejection (e.g., how often people exclude me or treat me unfairly) using an 8-item fixed-length form for people ages 18-85.
- Emotional Support: Assesses emotional support, referring to the perception that people in one’s social network are available to listen to one’s problems with empathy, caring, and understanding, using an 8-item fixed-length form for ages 18-85.
- Instrumental Support: Assesses instrumental support, referring to the perception that people in one’s social network are available to provide material or functional aid in completing daily tasks, using an 8-item fixed-length form for ages 18-85.
- Perceived Stress: Administered to ages 13-85 in CAT format, this measure assesses how unpredictable, uncontrollable, and overloaded respondents perceive their lives to be.
- Self-Efficacy: Administered to ages 8-85 in CAT format, this measure assesses respondents' sense of global self-efficacy, defined as a person’s belief in their capacity to manage functioning and have control over meaningful events.

NEO Five-Factor Inventory (NEO-FFI): This is the short-form version of the NEO Personality Inventory, designed to measure five core personality traits: Neuroticism, Extraversion, Openness to Experience, Agreeableness, and Conscientiousness. It consists of 60 items (12 per domain), derived from the original 240-item version (McCrae & Costa, 2004). Participants respond to self-descriptive statements using a Likert-type scale ranging from 1 (strongly disagree) to 5 (strongly agree). Each domain score is calculated by summing the responses to its 12 corresponding items. Importantly, 28 of these items are reverse-scored to account for variations in item direction.

Penn Emotion Recognition Test (ER40): This tool is widely used to assess the ability to recognize facial expressions of emotion. Participants are shown static images of human faces and asked to identify the emotion displayed in each image by selecting from a set of predefined options. The test evaluates accuracy in recognizing five fundamental emotions: anger, fear, happiness, neutrality, and sadness. The total number of correct responses for each emotion category is recorded (range 0-40). (Kohler et al., 2004).

Handedness: Participants' handedness was assessed using the Edinburgh Handedness Inventory (Oldfield, 1971). Scores range from -100 to 100, where negative values indicate a preference for left-handedness, and positive values indicate a preference for right-handedness.

Adult Self-Report (ASR): The ASR is a comprehensive self-assessment tool designed to evaluate various aspects of life functioning and psychological problems in adults aged 18 to 59. It includes multiple scales, such as Syndrome Scales and DSM-Oriented Scales, each serving distinct purposes in assessing an individual's mental health (<https://aseba.org/adults/>). This study included Syndrome Scales such as Anxious/Depressed, withdrawal, Somatic Complaints, Thought Problems, Aggressive Behavior, Rule-Breaking Behavior, and Intrusive. Additionally, we included DSM-Oriented Scales, specifically Depressive Problems, Anxiety Problems, Somatic Problems, Avoidant Personality Problems, and Antisocial Personality Problems.

The ASR comprises 126 items, each rated on a 3-point Likert scale (0 = Not True; 1 = Somewhat or Sometimes True; 2 = Very True or Often True). Responses are scored to generate T-scores for each scale, which are then compared to normative data to determine whether scores fall within normal, borderline, or clinical ranges (Guerrero et al., 2020).

Semi-Structured Assessment for the Genetics of Alcoholism (SSAGA): This is a comprehensive interview tool designed to assess psychiatric, behavioral, and substance use disorders based on DSM criteria (Bucholz et al., 1994). It includes modules covering a wide range of psychological and behavioral domains.

For this study, we specifically selected and analyzed the domains of Childhood Conduct Problems and Major Depressive Episodes to explore their relevance in understanding the relationship between psychological well-being and behavioral patterns.

**Section B: Statistical Analyses**

**Table S1.** Complete descriptive Statistics and Frequencies of the HCP Young Adult Cohort divided by cluster

| **Variable** | **PWB  Cluster** | | ***Mean (or frequencies)*** | ***Median*** | ***SD*** | ***IQR*** | ***Min*** | ***Max*** |
| --- | --- | --- | --- | --- | --- | --- | --- | --- |
| Male/Female | | Low | 63/75 | - | - | - | - | - |
|  | | Medium Low | 129/123 | - | - | - | - | - |
|  | | Medium High | 81/118 | - | - | - | - | - |
|  | | High | 40/63 | - | - | - | - | - |
| Age | | Low | 29.09 | 29 | 3.44 | 5.00 | 22 | 35 |
|  | | Medium Low | 29.03 | 29 | 3.57 | 6.00 | 22 | 36 |
|  | | Medium High | 29.09 | 29 | 3.50 | 6.00 | 22 | 36 |
|  | | High | 28.94 | 29 | 3.75 | 5.25 | 22 | 35 |
| Education (in years) | | Low | 14.93 | 16 | 1.78 | 2.00 | 11 | 17 |
|  | | Medium Low | 15.32 | 16 | 1.57 | 2.00 | 11 | 17 |
|  | | Medium High | 15.34 | 16 | 1.54 | 1.50 | 11 | 17 |
|  | | High | 14.99 | 16 | 1.75 | 2.00 | 11 | 17 |
| Single/In a Relationship | | Low | 91/47 | - | - | - | - | - |
|  | | Medium Low | 125/127 | - | - | - | - | - |
|  | | Medium High | 76/123 | - | - | - | - | - |
|  | | High | 28/68 | - | - | - | - | - |
| Handedness | | Low | 62.97 | 80 | 47.82 | 28.75 | -100 | 100 |
|  | | Medium Low | 67.96 | 80 | 38.82 | 30.00 | -95 | 100 |
|  | | Medium High | 69.15 | 80 | 43.43 | 30.00 | -100 | 100 |
|  | | High | 67.71 | 82.5 | 47.723 | 35.00 | -100 | 100 |
| MMSE | | Low | 29.09 | 29 | 0.95 | 1.00 | 26 | 30 |
|  | | Medium Low | 29.15 | 29 | 0.87 | 1.00 | 26 | 30 |
|  | | Medium High | 29.05 | 29 | 1.06 | 1.00 | 23 | 30 |
|  | | High | 29.00 | 29 | 1.02 | 2.00 | 27 | 30 |
| PSQI | | Low | 5.26 | 5 | 3.08 | 3.00 | 1 | 19 |
|  | | Medium Low | 4.45 | 4 | 2.25 | 3.00 | 0 | 15 |
|  | | Medium High | 4.11 | 4 | 2.41 | 3.00 | 0 | 12 |
|  | | High | 3.96 | 3 | 2.71 | 4.00 | 0 | 12 |
| Picture Sequence Memory Test | | Low | 110.98 | 110.70 | 13.00 | 16.50 | 76.42 | 135.55 |
|  | | Medium Low | 113.52 | 112.19 | 13.03 | 18.13 | 86.23 | 135.55 |
|  | | Medium High | 113.01 | 110.70 | 13.10 | 19.57 | 84.90 | 135.55 |
|  | | High | 114.06 | 113.72 | 11.46 | 16.87 | 93.23 | 135.55 |
| Dimensional Change Card Sort Test | | Low | 115.98 | 115.24 | 8.75 | 13.23 | 96.14 | 143.94 |
|  | | Medium Low | 115.08 | 114.32 | 9.59 | 12.04 | 85.37 | 143.94 |
|  | | Medium High | 116.04 | 116.50 | 11.04 | 14.18 | 81.27 | 143.94 |
|  | | High | 114.32 | 113.25 | 9.84 | 12.63 | 92.74 | 143.94 |
| Flanker Inhibitory Control and Attention Test | | Low | 112.00 | 111.54 | 9.81 | 11.32 | 86.31 | 142.11 |
|  | | Medium Low | 111.83 | 112.13 | 9.75 | 13.22 | 87.79 | 142.11 |
|  | | Medium High | 111.57 | 111.89 | 9.85 | 14.03 | 89.22 | 142.11 |
|  | | High | 111.98 | 112.30 | 9.65 | 13.03 | 92.88 | 135.02 |
| Oral Reading Recognition Test | | Low | 119.09 | 120.19 | 9.96 | 15.31 | 97.43 | 149.29 |
|  | | Medium Low | 117.79 | 118.68 | 10.22 | 13.74 | 88.30 | 150.71 |
|  | | Medium High | 118.54 | 119.13 | 9.77 | 12.93 | 91.32 | 150.71 |
|  | | High | 115.94 | 116.53 | 9.16 | 11.88 | 84.2 | 134.78 |
| Picture Vocabulary Test | | Low | 118.56 | 118.71 | 8.96 | 12.69 | 90.69 | 137.54 |
|  | | Medium Low | 118.61 | 117.50 | 8.55 | 11.68 | 98.60 | 145.27 |
|  | | Medium High | 118.12 | 118.21 | 8.39 | 11.59 | 99.33 | 145.27 |
|  | | High | 117.30 | 117.59 | 7.43 | 9.78 | 98.27 | 140.69 |
| Pattern Comparison Processing Speed Test | | Low | 116.15 | 116.24 | 14.02 | 16.55 | 60.09 | 154.69 |
|  | | Medium Low | 115.14 | 114.52 | 15.54 | 17.44 | 51.62 | 154.69 |
|  | | Medium High | 115.69 | 114.52 | 15.15 | 17.09 | 72.24 | 154.69 |
|  | | High | 117.54 | 114.99 | 15.80 | 25.11 | 89.64 | 151.8 |
| Delay Discounting_200 | | Low | 0.28 | 0.220 | 0.21 | 0.24 | 0.0157 | 0.98 |
|  | | Medium Low | 0.28 | 0.214 | 0.21 | 0.26 | 0.0195 | 0.98 |
|  | | Medium High | 0.28 | 0.232 | 0.21 | 0.25 | 0.0171 | 0.98 |
|  | | High | 0.29 | 0.226 | 0.21 | 0.27 | 0.0166 | 0.96 |
| Delay Discounting_40K | | Low | 0.56 | 0.575 | 0.26 | 0.44 | 0.0156 | 0.98 |
|  | | Medium Low | 0.55 | 0.563 | 0.28 | 0.45 | 0.0234 | 0.98 |
|  | | Medium High | 0.52 | 0.509 | 0.28 | 0.50 | 0.0171 | 0.98 |
|  | | High | 0.55 | 0.563 | 0.29 | 0.49 | 0.0156 | 0.98 |
| List Sorting Working Memory Test | | Low | 112.22 | 112.89 | 11.52 | 13.33 | 84.63 | 144.50 |
|  | | Medium Low | 112.78 | 112.89 | 10.93 | 14.83 | 84.63 | 144.50 |
|  | | Medium High | 113.68 | 112.89 | 10.92 | 13.83 | 84.63 | 144.50 |
|  | | High | 111.55 | 108.06 | 12.79 | 16.96 | 88.68 | 144.50 |
| Cognition Fluid Composite | | Low | 115.87 | 114.75 | 10.91 | 14.06 | 91.92 | 144.48 |
|  | | Medium Low | 116.20 | 114.92 | 10.79 | 15.26 | 86.68 | 144.67 |
|  | | Medium High | 116.76 | 115.69 | 11.52 | 18.01 | 93.04 | 144.86 |
|  | | High | 116.59 | 114.55 | 11.55 | 16.29 | 92.24 | 145.17 |
| Cognition Composite Score | | Low | 124.27 | 122.57 | 13.82 | 21.22 | 91.03 | 153.36 |
|  | | Medium Low | 123.84 | 123.44 | 13.22 | 19.68 | 95.86 | 153.36 |
|  | | Medium High | 124.61 | 122.51 | 13.78 | 19.18 | 96.56 | 153.36 |
|  | | High | 122.74 | 119.40 | 13.35 | 17.82 | 92.91 | 153.36 |
| Cognition Crystallized Composite | | Low | 119.88 | 120.14 | 9.29 | 13.16 | 94.81 | 143.13 |
|  | | Medium Low | 119.20 | 118.83 | 9.37 | 11.75 | 97.61 | 153.95 |
|  | | Medium High | 119.35 | 119.85 | 9.17 | 12.50 | 97.77 | 146.89 |
|  | | High | 117.52 | 117.37 | 8.00 | 11.93 | 92.95 | 140.81 |
| ER40_Anger | | Low | 6.87 | 7 | 1.02 | 2.00 | 4 | 8 |
|  | | Medium Low | 6.81 | 7 | 1.05 | 2.00 | 3 | 8 |
|  | | Medium High | 6.90 | 7 | 0.92 | 2.00 | 4 | 8 |
|  | | High | 6.66 | 7 | 1.14 | 1.25 | 2 | 8 |
| ER40_Fear | | Low | 6.92 | 7 | 1.17 | 2.00 | 1 | 8 |
|  | | Medium Low | 6.96 | 7 | 1.16 | 2.00 | 2 | 8 |
|  | | Medium High | 6.96 | 7 | 1.08 | 2.00 | 3 | 8 |
|  | | High | 6.71 | 7 | 1.41 | 2.00 | 0 | 8 |
| ER40_Happiness | | Low | 7.96 | 8 | 0.24 | 0.00 | 6 | 8 |
|  | | Medium Low | 7.96 | 8 | 0.20 | 0.00 | 7 | 8 |
|  | | Medium High | 7.96 | 8 | 0.18 | 0.00 | 7 | 8 |
|  | | High | 7.98 | 8 | 0.14 | 0.00 | 7 | 8 |
| ER40_Neutral | | Low | 7.20 | 8 | 1.15 | 1.00 | 3 | 8 |
|  | | Medium Low | 7.25 | 8 | 1.07 | 1.00 | 3 | 8 |
|  | | Medium High | 7.10 | 8 | 1.34 | 1.00 | 1 | 8 |
|  | | High | 7.09 | 7.5 | 1.29 | 1.00 | 0 | 8 |
| ER40_Sadness | | Low | 6.73 | 7 | 1.17 | 2.00 | 2 | 8 |
|  | | Medium Low | 6.81 | 7 | 1.09 | 2.00 | 3 | 8 |
|  | | Medium High | 6.95 | 7 | 0.98 | 2.00 | 4 | 8 |
|  | | High | 6.76 | 7 | 1.11 | 2.00 | 3 | 8 |
| Anger-Affect | | Low | 52.22 | 52.50 | 7.05 | 9.05 | 28.6 | 69.10 |
|  | | Medium Low | 47.86 | 48.40 | 6.82 | 7.55 | 28.6 | 67.90 |
|  | | Medium High | 45.57 | 45.70 | 7.30 | 9.45 | 28.6 | 66.90 |
|  | | High | 42.98 | 44.20 | 7.39 | 10.72 | 28.6 | 58.80 |
| Anger-Hostility | | Low | 55.30 | 57.35 | 7.92 | 9.45 | 36.6 | 71.00 |
|  | | Medium Low | 50.62 | 51.40 | 7.47 | 9.82 | 36.6 | 67.80 |
|  | | Medium High | 48.11 | 48.00 | 7.56 | 11.05 | 36.6 | 70.90 |
|  | | High | 44.47 | 43.05 | 7.14 | 12.32 | 36.6 | 63.10 |
| Anger-Physical Aggression | | Low | 52.47 | 52.20 | 8.22 | 14.07 | 43.4 | 81.40 |
|  | | Medium Low | 50.75 | 49.80 | 8.03 | 13.25 | 43.4 | 76.90 |
|  | | Medium High | 50.13 | 49.80 | 7.50 | 12.00 | 43.4 | 77.20 |
|  | | High | 48.97 | 43.40 | 7.49 | 9.05 | 43.4 | 76.30 |
| Fear-Affect | | Low | 54.59 | 54.00 | 8.00 | 9.45 | 32.9 | 74.60 |
|  | | Medium Low | 50.19 | 51.20 | 6.78 | 8.25 | 32.9 | 67.20 |
|  | | Medium High | 47.52 | 49.00 | 7.12 | 8.65 | 32.9 | 65.60 |
|  | | High | 46.51 | 48.00 | 6.89 | 8.50 | 32.9 | 66.90 |
| Fear-Somatic Arousal | | Low | 54.80 | 54.40 | 8.51 | 10.30 | 40.1 | 79.40 |
|  | | Medium Low | 51.82 | 52.45 | 7.69 | 11.10 | 40.1 | 76.40 |
|  | | Medium High | 51.22 | 50.50 | 7.95 | 12.05 | 40.1 | 76.40 |
|  | | High | 50.24 | 50.45 | 7.63 | 11.20 | 40.1 | 70.40 |
| Sadness | | Low | 53.14 | 53.30 | 7.12 | 9.30 | 34.2 | 71.60 |
|  | | Medium Low | 45.77 | 45.75 | 6.07 | 6.80 | 34.2 | 62.20 |
|  | | Medium High | 42.42 | 43.60 | 5.82 | 7.80 | 34.2 | 57.50 |
|  | | High | 40.80 | 41.30 | 5.58 | 10.55 | 34.2 | 56.30 |
| Friendship | | Low | 45.35 | 45.90 | 9.25 | 11.97 | 21.4 | 66.50 |
|  | | Medium Low | 49.97 | 49.80 | 7.91 | 9.25 | 21.9 | 66.50 |
|  | | Medium High | 53.00 | 52.50 | 7.35 | 9.15 | 35.7 | 66.50 |
|  | | High | 55.52 | 56.15 | 8.12 | 12.20 | 35.4 | 66.50 |
| Loneliness | | Low | 57.85 | 57.20 | 7.53 | 8.50 | 37.6 | 82.90 |
|  | | Medium Low | 50.72 | 52.00 | 7.10 | 7.00 | 37.6 | 73.00 |
|  | | Medium High | 47.36 | 47.40 | 6.71 | 8.90 | 37.6 | 62.10 |
|  | | High | 44.69 | 44.90 | 6.25 | 11.60 | 37.6 | 59.40 |
| Perceived Hostility | | Low | 50.53 | 51.40 | 8.62 | 9.97 | 33.5 | 72.50 |
|  | | Medium Low | 49.85 | 50.40 | 7.07 | 8.00 | 33.5 | 69.00 |
|  | | Medium High | 46.54 | 47.60 | 7.85 | 9.80 | 33.5 | 66.80 |
|  | | High | 44.42 | 44.35 | 8.45 | 12.82 | 33.5 | 62.50 |
| Perceived Rejection | | Low | 52.94 | 54.10 | 8.28 | 8.95 | 35.9 | 79.30 |
|  | | Medium Low | 49.36 | 51.40 | 7.43 | 9.80 | 35.9 | 72.40 |
|  | | Medium High | 44.94 | 44.50 | 7.68 | 15.75 | 35.9 | 63.70 |
|  | | High | 42.48 | 40.70 | 7.91 | 11.25 | 35.9 | 74.20 |
| Emotional Support | | Low | 44.99 | 43.90 | 9.90 | 12.75 | 20.1 | 62.50 |
|  | | Medium Low | 51.23 | 50.70 | 8.09 | 12.00 | 29.2 | 62.50 |
|  | | Medium High | 55.40 | 56.80 | 6.69 | 12.05 | 37.7 | 62.50 |
|  | | High | 58.47 | 62.50 | 5.98 | 5.90 | 37.5 | 62.50 |
| Instrumental Support | | Low | 44.24 | 44.75 | 8.77 | 11.15 | 22.1 | 62.90 |
|  | | Medium Low | 47.58 | 46.70 | 7.63 | 8.35 | 26.4 | 62.90 |
|  | | Medium High | 49.75 | 50.10 | 8.49 | 9.30 | 29.0 | 62.90 |
|  | | High | 53.71 | 54.00 | 8.50 | 16.20 | 30.0 | 62.90 |
| Perceived Stress | | Low | 54.75 | 54.75 | 8.09 | 10.60 | 33.3 | 75.00 |
|  | | Medium Low | 48.22 | 48.10 | 6.83 | 8.35 | 24.7 | 69.70 |
|  | | Medium High | 44.35 | 44.10 | 7.43 | 9.15 | 22.4 | 66.10 |
|  | | High | 41.13 | 41.65 | 7.61 | 11.00 | 22.4 | 59.00 |
| Self-Efficacy | | Low | 46.59 | 47.00 | 7.05 | 9.70 | 31.6 | 63.80 |
|  | | Medium Low | 49.63 | 48.00 | 6.83 | 8.52 | 33.9 | 68.40 |
|  | | Medium High | 53.49 | 53.00 | 7.83 | 10.70 | 37.0 | 68.40 |
|  | | High | 55.72 | 56.40 | 8.13 | 14.10 | 39.5 | 68.40 |
| Agreebleness | | Low | 32.24 | 32.50 | 5.27 | 6.75 | 13 | 44 |
|  | | Medium Low | 33.67 | 33 | 4.97 | 7.00 | 18 | 47 |
|  | | Medium High | 34.90 | 35 | 5.49 | 6.00 | 14 | 48 |
|  | | High | 36.04 | 35 | 5.69 | 8.00 | 17 | 46 |
| Oppenness to Experience | | Low | 28.93 | 28.5 | 6.16 | 9.00 | 15 | 45 |
|  | | Medium Low | 28.18 | 27.5 | 6.02 | 7.25 | 15 | 47 |
|  | | Medium High | 27.88 | 27 | 6.31 | 8.00 | 10 | 44 |
|  | | High | 27.30 | 27.5 | 6.17 | 8.00 | 13 | 44 |
| Conscientiousness | | Low | 31.60 | 32 | 5.77 | 8.00 | 11 | 45 |
|  | | Medium Low | 33.58 | 34 | 5.59 | 6.25 | 15 | 47 |
|  | | Medium High | 35.78 | 36 | 5.08 | 6.00 | 18 | 48 |
|  | | High | 36.78 | 37 | 6.32 | 8.25 | 12 | 48 |
| Neuroticism | | Low | 23.04 | 22.5 | 6.98 | 8.00 | 4 | 41 |
|  | | Medium Low | 16.43 | 16 | 5.96 | 7.25 | 1 | 34 |
|  | | Medium High | 13.50 | 13 | 6.51 | 7.50 | 0 | 38 |
|  | | High | 11.79 | 11 | 6.53 | 9.00 | 1 | 28 |
| Extraversion | | Low | 26.86 | 26.5 | 6.10 | 8.00 | 12 | 40 |
|  | | Medium Low | 30.80 | 31 | 5.21 | 6.00 | 11 | 44 |
|  | | Medium High | 32.47 | 33 | 5.24 | 6.00 | 15 | 45 |
|  | | High | 34.47 | 35 | 5.53 | 7.00 | 12 | 47 |
| Anxious/Depressed | | Low | 10.98 | 10 | 6.23 | 7.00 | 0 | 28 |
|  | | Medium Low | 5.34 | 4 | 4.30 | 5.00 | 0 | 22 |
|  | | Medium High | 3.69 | 3 | 3.24 | 4.00 | 0 | 16 |
|  | | High | 2.79 | 2 | 3.08 | 3.00 | 0 | 14 |
| Withdrawn | | Low | 3.71 | 3 | 2.68 | 4.00 | 0 | 12 |
|  | | Medium Low | 1.92 | 1 | 1.91 | 2.00 | 0 | 10 |
|  | | Medium High | 1.32 | 1 | 1.57 | 2.00 | 0 | 9 |
|  | | High | 0.88 | 0.5 | 1.11 | 1.00 | 0 | 4 |
| Somatic Complaints | | Low | 3.95 | 3 | 3.72 | 4.00 | 0 | 18 |
|  | | Medium Low | 2.27 | 1 | 2.62 | 4.00 | 0 | 16 |
|  | | Medium High | 1.73 | 1 | 2.06 | 2.50 | 0 | 12 |
|  | | High | 1.57 | 1 | 1.68 | 2.25 | 0 | 6 |
| Thought Problems | | Low | 2.76 | 2 | 2.30 | 3.00 | 0 | 13 |
|  | | Medium Low | 1.83 | 1 | 1.92 | 3.00 | 0 | 12 |
|  | | Medium High | 1.55 | 1 | 1.72 | 2.00 | 0 | 8 |
|  | | High | 1.25 | 1 | 1.48 | 2.00 | 0 | 7 |
| Aggressive Behavior | | Low | 5.20 | 5 | 3.77 | 4.75 | 0 | 18 |
|  | | Medium Low | 3.36 | 3 | 2.77 | 4.00 | 0 | 20 |
|  | | Medium High | 2.84 | 2 | 2.56 | 3.00 | 0 | 12 |
|  | | High | 2.21 | 2 | 2.00 | 2.25 | 0 | 8 |
| Rule Breaking Behavior | | Low | 2.96 | 2 | 2.80 | 4.00 | 0 | 14 |
|  | | Medium Low | 2.05 | 1.5 | 2.34 | 3.00 | 0 | 16 |
|  | | Medium High | 1.66 | 1 | 1.78 | 3.00 | 0 | 10 |
|  | | High | 1.23 | 1 | 1.56 | 2.00 | 0 | 7 |
| Intrusive | | Low | 2.10 | 2 | 1.94 | 3.00 | 0 | 7 |
|  | | Medium Low | 2.54 | 2 | 2.17 | 3.00 | 0 | 9 |
|  | | Medium High | 2.65 | 2 | 2.04 | 3.00 | 0 | 9 |
|  | | High | 2.38 | 2 | 2.56 | 4.00 | 0 | 11 |
| Depressive Problems | | Low | 7.02 | 7 | 4.35 | 5.00 | 0 | 19 |
|  | | Medium Low | 3.65 | 3 | 2.71 | 3.00 | 0 | 14 |
|  | | Medium High | 2.80 | 2 | 2.25 | 3.00 | 0 | 10 |
|  | | High | 2.33 | 2 | 2.02 | 2.00 | 0 | 9 |
| Anxiety Problems | | Low | 5.52 | 5 | 2.79 | 3.00 | 0 | 14 |
|  | | Medium Low | 3.65 | 3 | 2.43 | 3.00 | 0 | 11 |
|  | | Medium High | 3.05 | 3 | 2.18 | 3.00 | 0 | 11 |
|  | | High | 2.67 | 2 | 2.37 | 3.00 | 0 | 11 |
| Somatic Problems | | Low | 2.36 | 1.5 | 2.69 | 4.00 | 0 | 12 |
|  | | Medium Low | 1.44 | 1 | 1.92 | 2.00 | 0 | 11 |
|  | | Medium High | 1.01 | 1 | 1.43 | 1.00 | 0 | 10 |
|  | | High | 1.03 | 1 | 1.269 | 2.00 | 0 | 5 |
| Avoidant Personality Problems | | Low | 4.48 | 4 | 2.57 | 4.00 | 0 | 12 |
|  | | Medium Low | 2.67 | 2 | 1.99 | 3.00 | 0 | 9 |
|  | | Medium High | 1.90 | 2 | 1.75 | 3.00 | 0 | 8 |
|  | | High | 1.57 | 1 | 1.74 | 2.00 | 0 | 8 |
| Antisocial Personality Problems | | Low | 3.83 | 3 | 3.37 | 3.00 | 0 | 16 |
|  | | Medium Low | 2.63 | 2 | 2.60 | 3.00 | 0 | 15 |
|  | | Medium High | 2.23 | 2 | 2.12 | 2.00 | 0 | 13 |
|  | | High | 1.86 | 1.5 | 1.88 | 3.00 | 0 | 8 |
| Major Depressive Episode | | Low | 29 | - | - | - | - | - |
|  | | Medium Low | 17 | - | - | - | - | - |
|  | | Medium High | 13 | - | - | - | - | - |
|  | | High | 6 | - | - | - | - | - |

***Abbreviations:*** *SD, Standard Deviation; IQR, Interquartile Range; Min, Minimum; Max, Maximum; MMSE, Mini-Mental State Examination; PSQI, Pittsburgh Sleep Quality Index; ER, Emotion Recognition.*

***Table S2.*** *Participant's employment status*

| **Employment Status** |  | **PWB**  **Cluster** | **Frequencies** | **%  of Total** | **Cumulative %** |
| --- | --- | --- | --- | --- | --- |
| Not Working |  | Low | 21 | 3.1 % | 3.1 % |
|  |  | Medium Low | 23 | 3.4 % | 6.4 % |
|  |  | Medium High | 22 | 3.2 % | 9.6 % |
|  |  | High | 13 | 1.9 % | 11.5 % |
| Part-Time |  | Low | 20 | 2.9 % | 14.5 % |
|  |  | Medium Low | 38 | 5.5 % | 20.0 % |
|  |  | Medium High | 32 | 4.7 % | 24.7 % |
|  |  | High | 16 | 2.3 % | 27.0 % |
| Full-Time |  | Low | 97 | 14.2 % | 41.2 % |
|  |  | Medium Low | 191 | 27.9 % | 69.1 % |
|  |  | Medium High | 145 | 21.2 % | 90.2 % |
|  |  | High | 67 | 9.8 % | 100.0 % |

***Table S3.*** *Number of Childhood Conduct problems*

| **Childhood Conduct Problems** | | **PWB Cluster** | **Frequencies** | **% of Total** | **Cumulative %** | |
| --- | --- | --- | --- | --- | --- | --- |
| 0 |  | Low | 87 | 12.7 % | 12.7 % |  |
|  |  | Medium Low | 158 | 23.1 % | 35.8 % |  |
|  |  | Medium High | 123 | 18.0 % | 53.7 % |  |
|  |  | High | 68 | 9.9 % | 63.6 % |  |
| 1 |  | Low | 39 | 5.7 % | 69.3 % |  |
|  |  | Medium Low | 65 | 9.5 % | 78.8 % |  |
|  |  | Medium High | 55 | 8.0 % | 86.9 % |  |
|  |  | High | 23 | 3.4 % | 90.2 % |  |
| 2 |  | Low | 7 | 1.0 % | 91.2 % |  |
|  |  | Medium Low | 24 | 3.5 % | 94.7 % |  |
|  |  | Medium High | 18 | 2.6 % | 97.4 % |  |
|  |  | High | 2 | 0.3 % | 97.7 % |  |
| 3 |  | Low | 5 | 0.7 % | 98.4 % |  |
|  |  | Medium Low | 5 | 0.7 % | 99.1 % |  |
|  |  | Medium High | 3 | 0.4 % | 99.6 % |  |
|  |  | High | 3 | 0.4 % | 100.0 % |  |

***Note:*** *0=None; 1=1; 2=2, if male; 2=2 or more, if female; 3=3 or more, if male*

**References**

Bucholz, K. K., Cadoret, R., Cloninger, C. R., Dinwiddie, S. H., Hesselbrock, V. M., Nurnberger, J. I., Reich, T., Schmidt, I., & Schuckit, M. A. (1994). A new, semi-structured psychiatric interview for use in genetic linkage studies: a report on the reliability of the SSAGA. *Journal of Studies on Alcohol*, *55*(2), 149–158. https://doi.org/10.15288/JSA.1994.55.149

Buysse, D. J., Ancoli-Israel, S., Edinger, J. D., Lichstein, K. L., & Morin, C. M. (2006). Recommendations for a Standard Research Assessment of Insomnia. *Sleep*, *29*(9), 1155–1173. https://doi.org/10.1093/SLEEP/29.9.1155

da Matta, A., Gonçalves, F. L., & Bizarro, L. (2012). Delay discounting: concepts and measures. *Psychology & Neuroscience*, *5*(2), 135–146. https://doi.org/10.3922/J.PSNS.2012.2.03

Folstein, M. F., Folstein, S. E., & McHugh, P. R. (1975). “Mini-mental state”: A practical method for grading the cognitive state of patients for the clinician. *Journal of Psychiatric Research*, *12*(3), 189–198. https://doi.org/10.1016/0022-3956(75)90026-6

Foy, J. G., & Foy, M. R. (2020). Dynamic Changes in EEG Power Spectral Densities During NIH-Toolbox Flanker, Dimensional Change Card Sort Test and Episodic Memory Tests in Young Adults. *Frontiers in Human Neuroscience*, *14*, 526305. https://doi.org/10.3389/FNHUM.2020.00158

Galasko, D., Lasker, B., Thal, L. J., Klauber, M. R., Salmon, D. P., & Hofstetter, C. R. (1990). The Mini-Mental State Examination in the Early Diagnosis of Alzheimer’s Disease. *Archives of Neurology*, *47*(1), 49–52. https://doi.org/10.1001/ARCHNEUR.1990.00530010061020

Guerrero, M., Hoffmann, M., & Pulkki-Råback, L. (2020). Psychometric Properties of the Adult Self-Report: Data from over 11,000 American Adults. *Stats 2020, Vol. 3, Pages 465-474*, *3*(4), 465–474. https://doi.org/10.3390/STATS3040029

Halcomb, M. (2023). The Delay Discounting Procedure: Methodology and Flexibility. *Neuromethods*, *201*, 35–54. https://doi.org/10.1007/978-1-0716-3267-3_3

Kohler, C. G., Turner, T. H., Gur, R. E., & Gur, R. C. (2004). Recognition of Facial Emotions in Neuropsychiatric Disorders. *CNS Spectrums*, *9*(4), 267–274. https://doi.org/10.1017/S1092852900009202

Loring, D. W., Bowden, S. C., Staikova, E., Bishop, J. A., Drane, D. L., & Goldstein, F. C. (2019). NIH Toolbox Picture Sequence Memory Test for Assessing Clinical Memory Function: Diagnostic Relationship to the Rey Auditory Verbal Learning Test. *Archives of Clinical Neuropsychology*, *34*(2), 268–276. https://doi.org/10.1093/ARCLIN/ACY028

McCrae, R. R., & Costa, P. T. (2004). A contemplated revision of the NEO Five-Factor Inventory. *Personality and Individual Differences*, *36*(3), 587–596. https://doi.org/10.1016/S0191-8869(03)00118-1

Myerson, J., Green, L., & Warusawitharana, M. (2001). AREA UNDER THE CURVE AS A MEASURE OF DISCOUNTING. *Journal of the Experimental Analysis of Behavior*, *76*(2), 235–243. https://doi.org/10.1901/JEAB.2001.76-235

Oldfield, R. C. (1971). The assessment and analysis of handedness: The Edinburgh inventory. *Neuropsychologia*, *9*(1), 97–113. https://doi.org/10.1016/0028-3932(71)90067-4

Rueda, M. R., Fan, J., McCandliss, B. D., Halparin, J. D., Gruber, D. B., Lercari, L. P., & Posner, M. I. (2004). Development of attentional networks in childhood. *Neuropsychologia*, *42*(8), 1029–1040. https://doi.org/10.1016/J.NEUROPSYCHOLOGIA.2003.12.012

Weintraub, S., Dikmen, S. S., Heaton, R. K., Tulsky, D. S., Zelazo, P. D., Bauer, P. J., Carlozzi, N. E., Slotkin, J., Blitz, D., Wallner-Allen, K., Fox, N. A., Beaumont, J. L., Mungas, D., Nowinski, C. J., Richler, J., Deocampo, J. A., Anderson, J. E., Manly, J. J., Borosh, B., … Gershon, R. C. (2013). Cognition assessment using the NIH Toolbox. *Neurology*, *80*(11 Suppl 3). https://doi.org/10.1212/WNL.0B013E3182872DED
